# Fragment-based screening identifies novel targets for inhibitors of conjugative transfer of antimicrobial resistance by plasmid pKM101

**DOI:** 10.1038/s41598-017-14953-1

**Published:** 2017-11-02

**Authors:** Bastien Casu, Tarun Arya, Benoit Bessette, Christian Baron

**Affiliations:** Department of Biochemistry and Molecular Medicine, Faculty of Medicine, Université de Montréal, 2900 Boulevard Édouard-Montpetit, Montreal, QC H3T 1J4 Canada

## Abstract

The increasing frequency of antimicrobial resistance is a problem of global importance. Novel strategies are urgently needed to understand and inhibit antimicrobial resistance gene transmission that is mechanistically related to bacterial virulence functions. The conjugative transfer of plasmids by type IV secretion systems is a major contributor to antimicrobial resistance gene transfer. Here, we present a structure-based strategy to identify inhibitors of type IV secretion system-mediated bacterial conjugation. Using differential scanning fluorimetry we screened a fragment library and identified molecules that bind the essential TraE protein of the plasmid pKM101 conjugation machinery. Co-crystallization revealed that fragments bind two alternative sites of the protein and one of them is a novel inhibitor binding site. Based on the structural information on fragment binding we designed novel small molecules that have improved binding affinity. These molecules inhibit the dimerization of TraE, bind to both inhibitor binding sites on TraE and inhibit the conjugative transfer of plasmid pKM101. The strategy presented here is generally applicable for the structure-based design of inhibitors of antimicrobial resistance gene transfer and of bacterial virulence.

## Introduction

The conjugative transfer of plasmids between Gram-negative bacteria is mediated by type IV secretion system (T4SS) and many pathogens such as *Helicobacter pylori*, *Bartonella* and *Brucella* species use this strategy for the transfer of virulence factors into mammalian cells. T4SS typically comprise 12 membrane-associated proteins that fall into three categories: cytoplasmic ATPases (VirB4, VirB11 and VirD4), surface-exposed pilus components (VirB2 and VirB5) and core components associated with the inner and/or the outer membrane (VirB1, VirB3, VirB6, VirB7, VirB8, VirB9 and VirB10)^1,2^. X-ray and NMR structures of several individual proteins are available and additional information on the overall T4SS complex structure was obtained by electron microscopy and X-ray crystallography of subcomplexes from plasmid conjugation systems pKM101 and R388^3–5^. Considering the importance of T4SS for bacterial virulence and for the transfer of antimicrobial resistance (AMR) genes^1,2,6^, it would be interesting to design inhibitors of this process and these molecules could also serve as chemical probes for mechanistic studies^7–9^. Small molecule inhibitors of the ATPase activity of VirB11 homolog Cagα that inhibit the virulence of *H*. *pylori* were described, but no structural information on their binding site is available^10^. Inhibitors of *Brucella* VirB8 were identified using an *in vivo* dimerization assay based on the bacterial two–hybrid system^11^. X-ray analysis of the periplasmic domain of the *Brucella* VirB8 protein, co-crystallized with an inhibitor, and *in silico* docking showed that these molecules bind to a surface groove of the protein^12^. The most active VirB8 inhibitors are salicylidene acylhydrazides that inhibit dimerization of the protein and also the virulence of *Brucella* ^12^. Previous work has shown that derivatives of the most active molecule B8I-2 bind to the VirB8 homolog TraE from pKM101, inhibit its dimerization and conjugative transfer of the plasmid^13^. Interestingly, the predicted inhibitor binding site is distant from the dimerization site of the protein, and until now is not clear how these molecules inhibit dimerization and protein functions^14^. Here, we have identified novel chemical entities that bind to the previously known site as well as to a novel site close to the dimerization interface of the protein.

## Results

### Screening for TraE-binding fragments

**Figure 1:**
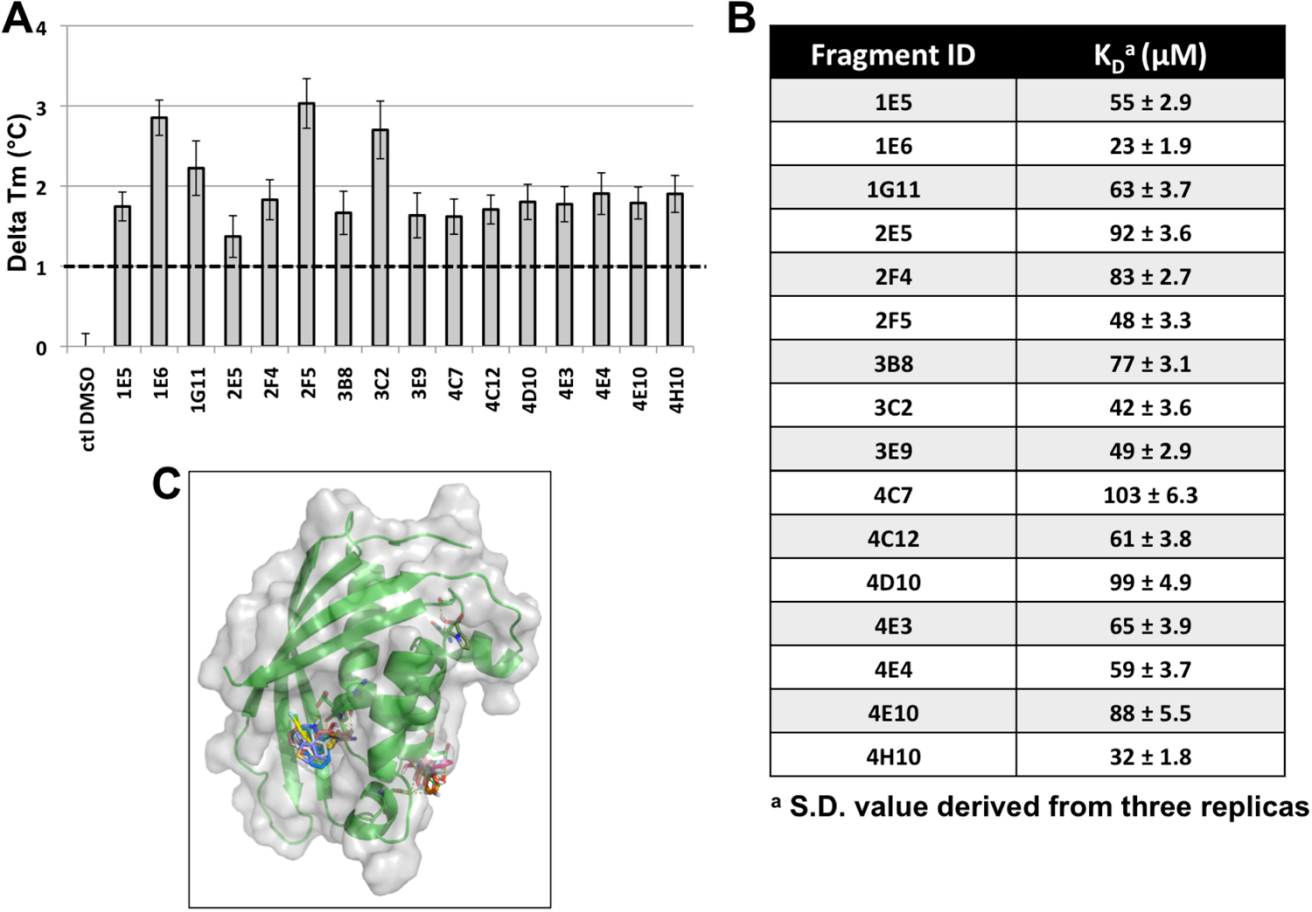
Binding fragments identified by DSF screening. (**A**) Replicates of DSF experiment for binding fragments. Data presented are from three separate experiments conducted in triplicates, error bars reflect the standard deviation. (**B**) Predicted binding of fragments to TraE using AutoDockVina. (**C**) Binding of fragments to TraE calculated using fluorescence quenching as assay.

Here, we conducted a fragment-based screen to identify novel chemical entities that bind to and may identify novel druggable sites on TraE and other VirB8-like proteins. Fragment libraries are smaller (usually a few hundred molecules) than the libraries of small molecules typically used for high-throughout screening (several thousand to millions) and the screens can generally be conducted in a standard laboratory setting without highly specialized equipment^15,16^. The binding affinities of fragments that are typically smaller than 300 Da and lower than that of small molecules (larger than 500 Da), but fragments are excellent starting points for the synthesis of more potent small molecules contributing to the increasing popularity of fragment-based drug discovery (FBDD)^16^. Using a differential scanning fluorimetry (DSF) assay in an RT-PCR instrument we screened a fragment library of 505 molecules (supplementary Fig. 1) identifying 16 molecules that significantly increase the thermal melting point of the purified periplasmic domain of TraE (Fig. 1A). The structures of the molecules are diverse, yet all but one contain a carboxyl group that may be involved in interactions with charged amino acid side chains of the target (supplementary Fig. 2). Fluorescence quenching shows binding of the fragments and the K_D_ values between 23 μM and 103 μM are in the range of what is expected for fragments (Fig. 1B)^16^.

### Identification of the binding sites on TraE and design of small molecule derivates

**Figure 2:**
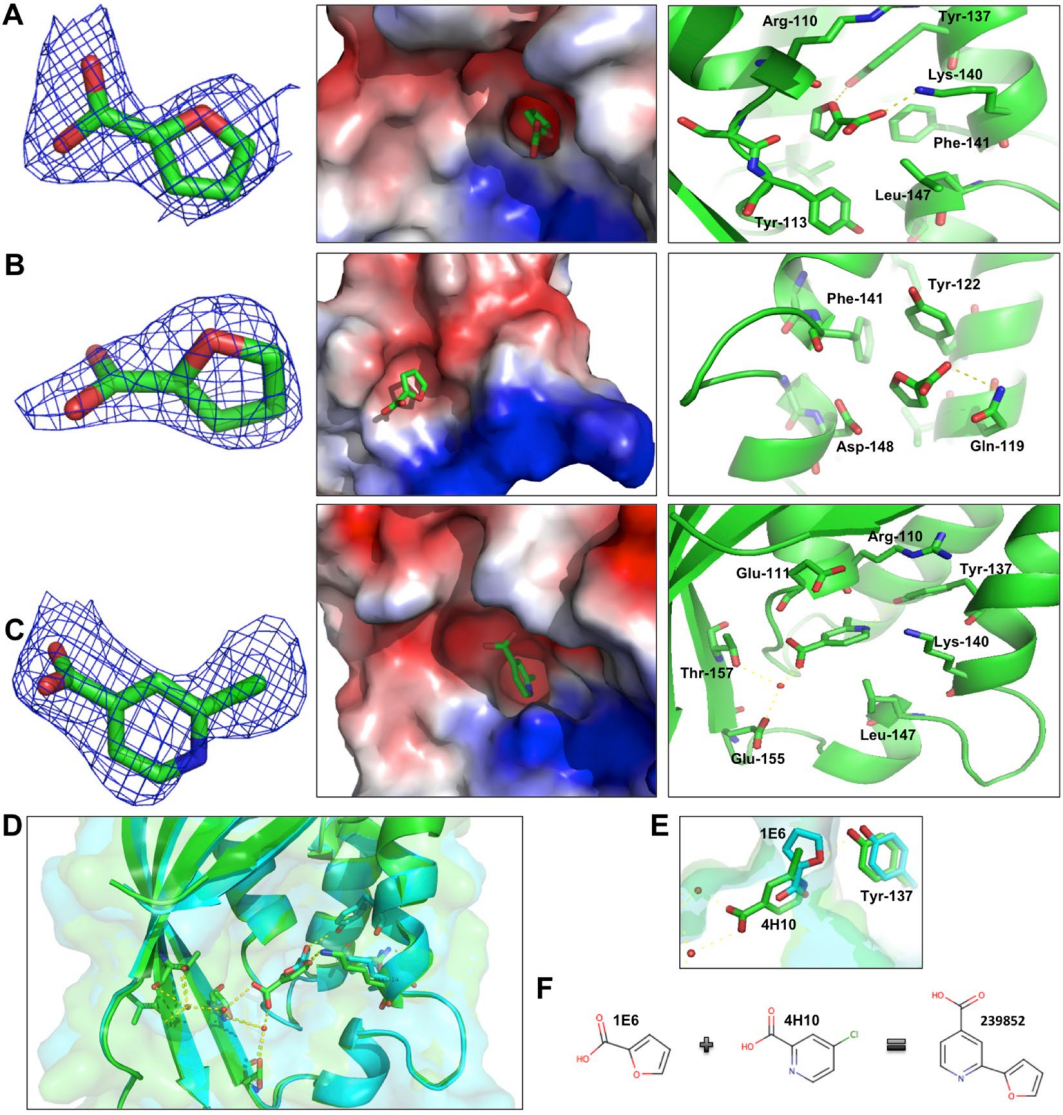
X-ray crystal structures of fragments bound to TraE. (**A**) First binding site of 1E6 fragment, (**B**) second binding site of 1E6 fragment and (**C**) 4H10 fragment bound to TraE. From left to right, fragment stick representations with the 2Fo-Fc electron density maps shown as a blue mesh at ≥ 1.0 σ, electrostatic potential surface representations of TraE with fragments inside the binding pockets and amino acids in the binding pocket of each fragment. (**D**) Overlay of the TraE-1E6 (in green) and TraE-4H10 (in blue) complex crystal structures (RMSD is 0.69 Å) with the description of the amino acids participating in hydrogen bonds represented by dashed yellow lines. Hydrogen bonds with fragments are represented by dashed yellow lines. Water molecules coordinating binding between the fragments and TraE are shown as small red spheres. (**E**) Focus on the position of fragments 1E6 and 4H10 inside the binding pocket **(F**) Schematic combination of fragments 1E6 and 4H10 binding to TraE to design small molecule inhibitor 239852.

*In silico* docking suggests that many fragments may bind to a surface groove of TraE that was already shown to be an inhibitor binding site in VirB8-like proteins^12,13^. In addition, docking predicted an additional potential binding site in an α-helical region of TraE that is close to the dimerization interface (Fig. 1C). Whereas docking predictions are increasingly reliable, the design of inhibitors requires high-resolution structural information and towards this goal we soaked TraE crystals with the binding fragments. We obtained X-ray structures in case of two fragments (1E6 and 4H10) that diffracted to 2.5 Å and 2.79 Å resolution, respectively (supplementary Table 2). Surprisingly, molecule 1E6 (2-furoic acid) bound to two sites on TraE (Fig. 2A and B). The first binding site is in the inhibitor-binding surface groove, whereas the second molecule binds to an α-helical region at the dimerization site of VirB8-like proteins as was predicted by docking. In contrast, molecule 4H10 (2-chloroisonicotinic acid) binds adjacently to the first binding site of molecule 1E6 (Figs. 2D and E). Since both fragments bind in close proximity we reasoned that combining them may generate small molecules with higher affinity. Based on this rationale we obtained molecule 239852 (2-(2-furyl)isonicotinic acid) (Fig. 2F), which is essentially a fusion of 4H10 and 1E6, as well as four other similar molecules from a commercial supplier (Enamine).

### Small molecules binding two alternative sites in TraE

**Figure 3:**
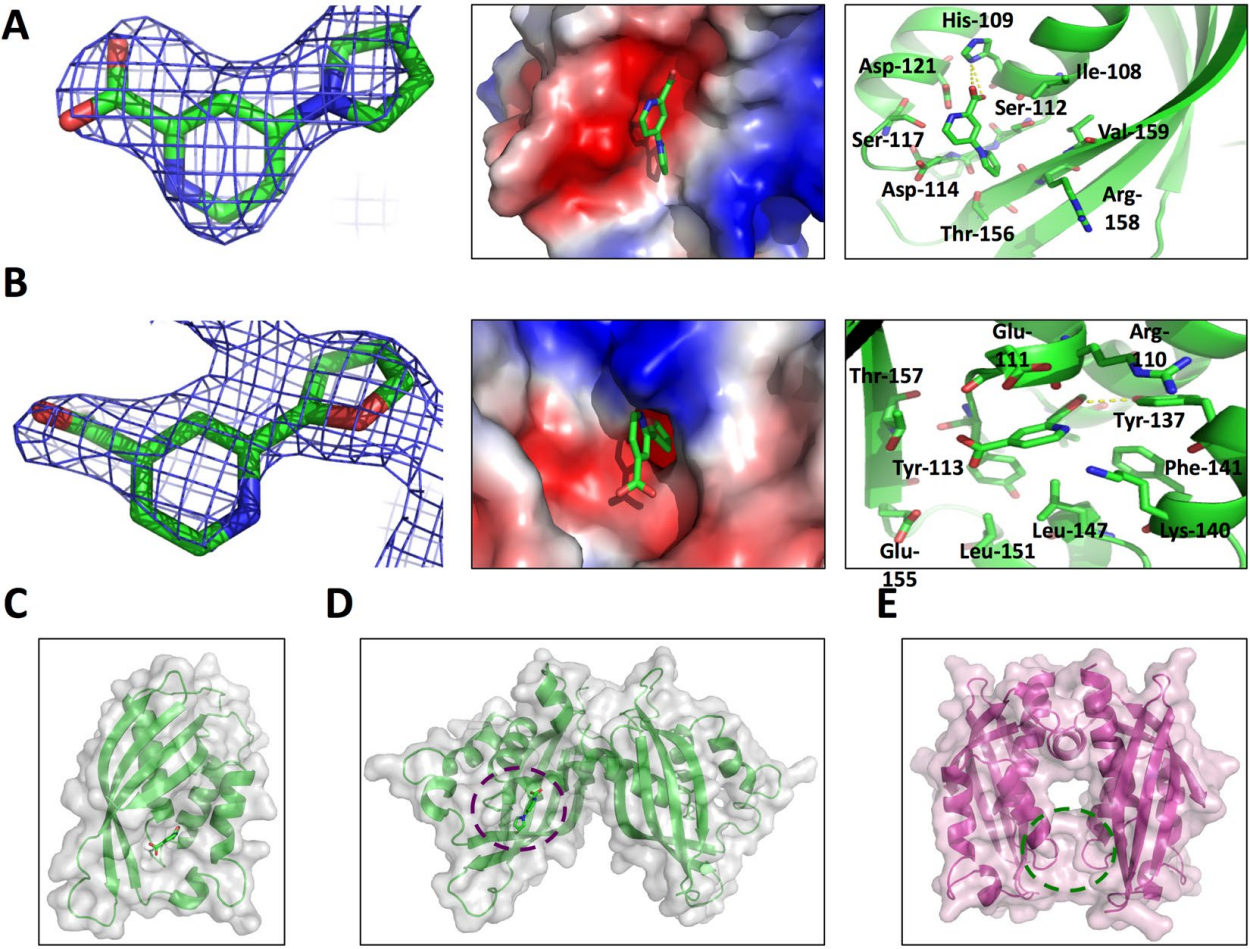
X-ray crystal structure of small molecules bound to TraE. (**A**) Binding sites of molecule 105055 and (**B**) of molecule 239852. From left to right, fragment stick representations with the 2Fo-Fc electron density maps shown as a blue mesh at 0.9 σ for 239852 and 1.0 σ for 105055, electrostatic potential surface representations of TraE-bound small molecules and amino acids implicated in binding, hydrogen bonds with small molecules are represented by dashed yellow lines. (**C**) Overall geometry of TraE structure in complex with molecule 239852 (**D**,**E**). Overall geometry of TraE dimer structure in complex with molecule 105055 and comparison with the structure of the VirB8 dimer from *Brucella suis* (PDB code 2BHM)^20^. The position of the binding site is represented by a dashed purple circle for TraE and by a dashed green circle for VirB8.

*In silico* docking predicted that all these molecules bind to one or both of the 1E6 binding sites observed by X-ray crystallography (supplementary Fig. 3). Analysis by X-ray crystallography revealed that two of the molecules bind to the two alternative sites on TraE thus confirming the docking predictions. Molecule 239852 binds to the previously described inhibitor binding surface groove (Fig. 3A and C), whereas molecule 105055 (4-(1H-pyrrol-1-yl)pyridine-2-carboxylic acid) binds to the α-helical region of TraE close to the dimerization site of VirB8-like molecules (Fig. 3B and D). Interestingly, X-ray structure analysis of TraE revealed that the overall geometry of the dimer (Fig. 3D) differs from that of other VirB8 homologs (Fig. 3E)^13^. This difference may reflect two conformational states of VirB8-like proteins. This opens the possibility that molecule 105055 may directly interfere with dimerization, while molecule 239852 could exert an effect similar to previously described VirB8 inhibitors.

### Novel small molecules bind TraE and inhibit its functions

**Figure 4:**
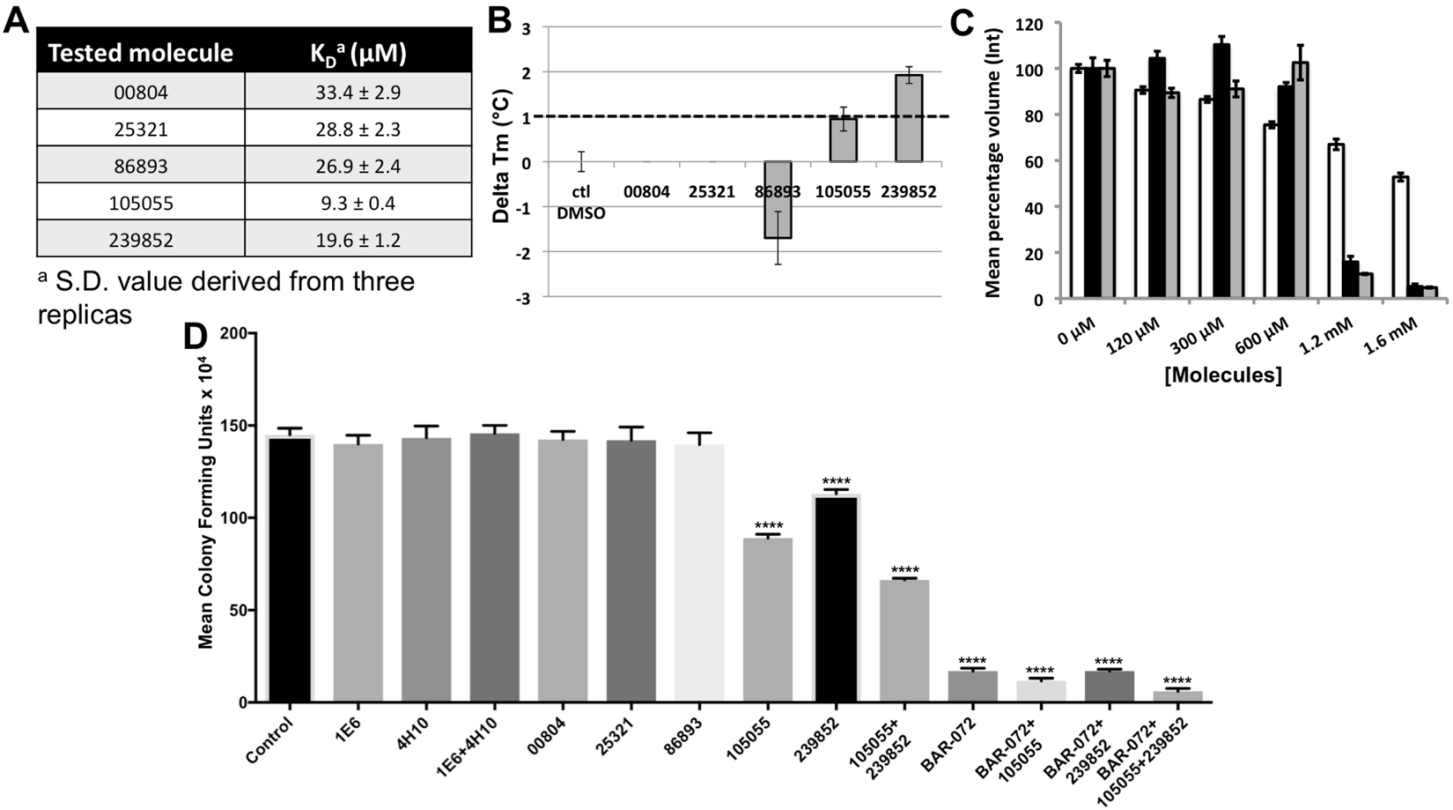
Characterization of the effects of small molecules *in vitro* and *in vivo*. (**A**) Binding of small molecules to TraE calculated using the fluorescence quenching assay. (**B**) DSF experiments to assess binding of small molecules. Data presented are from three separate experiments conducted in triplicates, error bars reflect the standard deviation. (**C**) Formation of DSS-dependent cross-linking products of TraE in the absence and in the presence (0–1.6 mM) of molecules 239852, 105055, and combination of 105055 and 239852. Formation of the cross-linking product was normalized by comparison with the control without small molecules (100% is the control), shown in white for 239852, in black for 105055 and in grey for the combination of 105055 and 239852; the data represent averages and S.E. (error bars) of the mean of three replicates. (**D**) Conjugation assays between pKM101-carrying donor strain FM433 and plasmid-free recipient WL400 in the presence of 50 μM TraE-binding fragments and small molecules. The numbers of colony-forming units compared with a control experiment in the absence of the small molecules are shown; data represent averages and S.E. (error bars) of three biological replicate cultures. Asterisks, statistically significant differences (p ≤ 0.0001, n = 3).

To assess whether the 4H10/1E6-derived molecules bind TraE and impact protein function we next conducted a series of *in vitro* and *in vivo* assays. First, we tested binding to TraE using fluorescence quenching, revealing that both molecules 105055 and 239852 have increased effects as compared to the original fragments 4H10/1E6 and they also stabilized the protein as assessed by DSF (Fig. 4A and B). The other derived molecules have binding activities similar to the original fragments, but they do not induce positive thermal shift changes, suggesting that their interaction with TraE is relatively weak (Fig. 4A and B). Second, we tested the effects of molecules 105055 and 239852 on dimerization using an *in vitro* cross-linking assay with the purified periplasmic domain of TraE. Both molecules significantly inhibit cross-linking, inhibition is more pronounced in the case of molecule 105055 and combination of both molecules further accentuates inhibition (Fig. 4C and supplementary Fig. 4). Third, we analysed whether the original fragments (4H10 and 1E6) or the derived molecules inhibit the conjugative transfer of pKM101. Addition of the fragments as well as of the derived small molecules that do not stabilize the protein in the DSF assay at concentrations of 50 μM has no effect on conjugation. However, molecules 105055 as well as 239852 significantly reduce conjugative DNA transfer (Fig. 4D). The efficacy of conjugative transfer is further decreased to about 45% of the control when both molecules are combined. Interestingly, combination with the known TraE inhibitor BAR-072^13^ further reduces conjugation of these molecules to values as low as 4% of the control (Fig. 4D). The molecules do not negatively impact bacterial viability and they have no effect on transfer of the unrelated plasmid RP4, suggesting that the effect is specific for pKM101 (supplementary Fig. 5,^13^).

## Discussion

We here present a generally applicable structure-based strategy to identify inhibitors of bacterial conjugation. The DSF approach used as primary assay to screen a library of several hundred fragments can be conducted in most molecular biology laboratories since it uses a commonly available RT-PCR instrument and does not necessitate specialized high-throughput screening equipment. Using this approach we confirmed that the previously identified inhibitor-binding surface groove of VirB8-like proteins is indeed a promising target for the binding of inhibitors that could be further explored for drug design. Despite repeated efforts in our group (^12–14^ and unpublished work) we have obtained only one co-crystal structure with a binding small molecule (B8I-1) that did not have a strong effect on *Brucella* virulence^11^. All the data on the binding of the most biologically active molecules (salicylidene acylhydrazide B8I-2 and its derivatives) to VirB8 and TraE were obtained by *in silico* docking – an approach that is increasingly reliable, but that cannot replace actual structural information. For this reason, it was very important to confirm by X-ray crystallography that two of the identified fragments (4H10 and 1E6) indeed bind to the surface groove of the protein that was predicted to bind B8I-2 and its derivates. To our surprise, molecule 1E6 also binds to an alternative site that is close to the dimer interface of VirB8-like proteins. The discovery of this novel binding site opens additional opportunities for the design of small molecules that inhibit the functions of VirB8-like proteins.

Since fragments 4H10 and 1E6 bind adjacently to the surface groove, we designed a series of small molecules that essentially represent a fusion of these molecules. Interestingly, the newly designed small molecules 105055 as well as 239852 bind to both respective sites that have been identified by fragment screening. Even more importantly, they inhibit dimerization of the protein as well as conjugative transfer of plasmid pKM101. The degree of inhibition is relatively modest, but it is further enhanced by combining the two molecules also adding the previously identified conjugation inhibitor BAR-072^13^. This work constitutes a key step for the structure-based design of more potent conjugation inhibitors based on the novel chemical entities we have identified here. Also, our data demonstrate the existence of two potential druggable sites on TraE. It will be interesting to assess whether both sites can be exploited for fragment-based approaches aimed at identifying inhibitors of other VirB8-like proteins.

The strategy validated here is applicable to VirB8-like proteins from the T4SS of bacterial pathogens for which structural and functional information is available^17–20^. However, the application is not limited to T4SS components and this approach could be broadly applied to any conjugation system component or virulence factor for which high-resolution structural information is available. The availability of a defined target and of structural information is crucial for structure-based drug design aimed at improving hits identified by screening. In the absence of such information on the target and its structure, it can be quite challenging to further advance inhibitor and drug development. This was the case for inhibitors of bacterial type III secretion systems where it took several years after discovery of the molecules to identify the targets and the inhibitor binding sites^9,21^. The strategy presented here can be applied to a wide variety of virulence factors and has potential to contribute to the development of drugs that reduce AMR gene transfer and bacterial virulence.

## Methods

### Protein overexpression and purification

Protein overexpression and purification was performed as described previously^13^. The TraE buffer was changed to 20 mM HEPES, 50 mM NaCl, pH 7.4. TraE protein concentrations were determined using the molar extinction coefficient at 280 nm of 25 900 M^−1^ cm^−1^.

### Differential scanning fluorimetry

A library of 505 fragments was used in this study of which 186 were previously described^14^ and the others are listed in Supplementary Table 3. DSF experiments were performed as described previously^14^. DSF was conducted using 5 μM of protein, 10X concentration of SYPRO Orange (from 5000X stock solution, ThermoFisher) in 20 mM HEPES, 50 mM NaCl, pH 7.4 and 5% final concentration of DMSO. For fragment-based screening and molecule optimization, fragments or small molecules were added to final concentrations of 5 mM. SYPRO Orange fluorescence was monitored over 20–95 °C with a LightCycler® 480 instrument (Roche, USA). The LightCycler® 480 Software (Roche) was used to calculate the first derivate of the resulting melting curve, with the steepest point of the slope being the Tm. Fragments were considered to be hits when ΔT_m_ was higher than 1 °C, which is two times higher than the standard deviation of the DMSO control.

### Analysis of small molecule binding by fluorescence spectroscopy

Changes of the intrinsic UV fluorescence emission of TraE upon binding of fragments and small molecules were recorded at 20 °C with a Cary Eclipse Fluorometer (Varian) (λ_ex_ 295 nm, λ_em_ 340 nm, 5 nm excitation and emission slit widths) in 20 mM HEPES, 50 mM NaCl, pH 7.4. The spectra were corrected for dilution effects and K_D_ values were calculated from the ligand binding fluorescence data fitted to a single site saturation curve with constant background using the Grafit 6.0 software package.

### Crystallization of TraE, formation of fragment or small molecule complexes with TraE crystals and data collection

The TraE protein (15 mg/ml) was crystallized in 16% (w/v) PEG 10 000, 50 mM Bis-Tris (pH 5.5), 100 mM ammonium acetate. To obtain TraE−fragment or TraE-small molecule complexes, crystals of TraE were soaked for time periods ranging from 1 min to 6 h in a cryoprotectant solution containing 16% (w/v) PEG 10 000, 50 mM Bis-Tris (pH 5.5), 100 mM ammonium acetate, 20% ethylene glycol and 1−10 mM fragments or small molecules. After soaking, the crystals were flash frozen in liquid nitrogen and the data were collected at the Cornell High Energy Synchrotron Source (CHESS, USA) beamline F1. The intensity data was processed using the HKL2000 program.

### Structure determination of TraE

Structures were solved by molecular replacement using the template (PDB # 5I97) as reference model. Refinement was performed using Phenix software suites to achieve the highest possible resolution^22^. Electron density maps were calculated to the resolution indicated in supplementary Table 2. Final model statistics, calculated with Phenix, molprobity and PROCHECK, are shown in supplementary Table 2. Stereochemical restraints for ligands were generated with Elbow from Phenix software. The atomic coordinates and structure factors for TraE in complex with fragments and small molecules have been deposited at the Protein Data Bank (PDB # 5WIC, PDB # 5WII, PDB # 5WIO, PDB # 5WIP). All figures were prepared using the program PyMOL.

### Molecular docking analysis

*In silico* docking was performed using Autodock Vina^23^ run through PyRx to manage the workflow and PyMol to visualize the results. The chemical structures for each ligand were drawn with MarvinSketch (v15.7.13.0, 2015), ChemAxon (http://www.chemaxon.com), converted to PDB format, followed by processing with Autodock Tools 1.5.4 to assign Gasteiger charges, merging non-polar hydrogens and to set torsional bonds. Docking runs were performed within a 47 × 52 × 33 Å rectangle search space surrounding the binding pocket, and output modes were ranked according to binding affinity (BA).

### Analysis of protein-protein interactions by crosslinking

Chemical crosslinking with disuccinimidyl suberate (DSS, Pierce) was performed as described previously^13^ and the formation of crosslinking products was quantified by SDS-PAGE and using the ImageLab Software 4.0 (Bio-Rad).

### Quantitation of conjugative DNA transfer

Quantitation of conjugative DNA transfer was performed as described previously^13^. For analysis of the inhibition of conjugation by fragments or small molecules, the cells were cultivated on agar and in liquid media in the presence of 50 μM of the small molecules in the presence of 2.5% DMSO. The presence of DMSO and of the fragments or small molecules did not negatively impact bacterial growth.

## Electronic supplementary material

Supplementary information
