# Supplementary material for: Fragment-based screening identifies novel targets for inhibitors of conjugative transfer of antimicrobial resistance by plasmid pKM101
Source: Sci Rep. 2017 Nov 2;7:14907. doi: 10.1038/s41598-017-14953-1 (PMC5668240; doi:10.1038/s41598-017-14953-1)
Supplement: Supplementary file 1 — Supplementary information [file 41598_2017_14953_MOESM1_ESM.pdf]

Title : **Fragment-based screening identifies novel targets for inhibitors of conjugative transfer of antimicrobial resistance by plasmid pKM101**

Authors : Bastien Casu, Tarun Arya, Benoit Bessette, Christian Baron*

Affiliation : Department of Biochemistry and Molecular Medicine, Faculty of Medicine, Université de Montréal, 2900 Boulevard Édouard-Montpetit, Montréal, QC, H3T 1J4, Canada

***Corresponding Author:** Christian Baron

Université de Montréal, 2900 Boulevard Édouard-Montpetit, Montréal, QC, H3T 1J4, Canada

E-mail: christian.baron@umontreal.ca

Phone: (514) 343-6300

Supplementary Figure 1

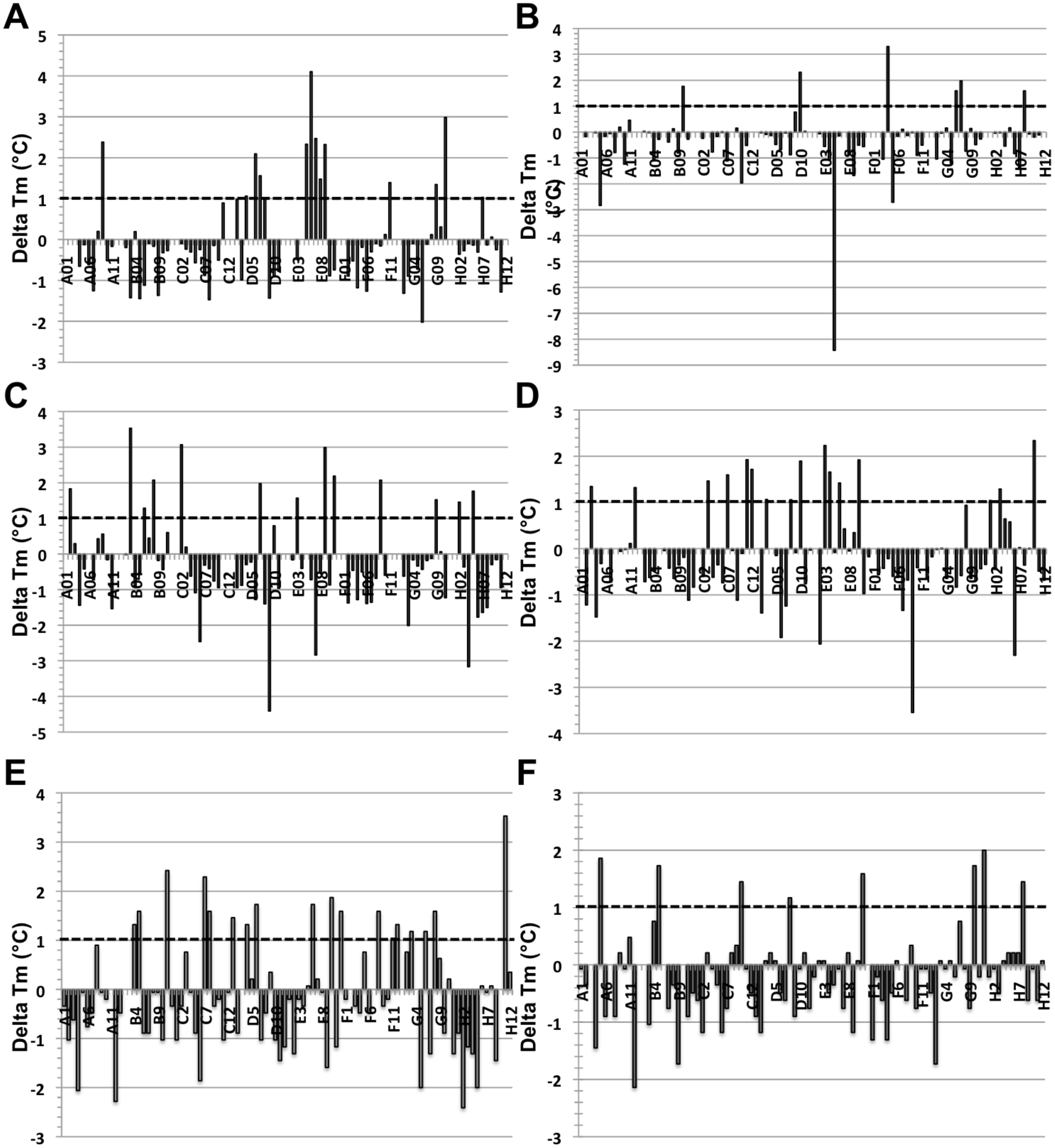

Supplementary Fig. 2

Name	Structure	Name	Structure	Name	Structure	Name	Structure
1E5	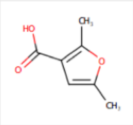	2F4	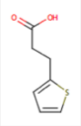	3E9	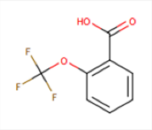	4E3	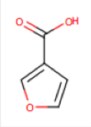
1E6	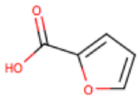	2F5	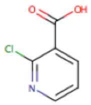	4C7	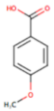	4E4	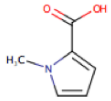
1G11	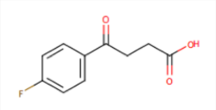	3B8	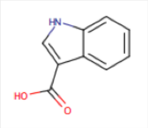	4C12	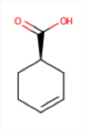	4E10	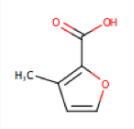
2E5	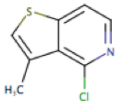	3C2	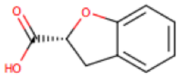	4D10	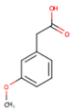	4H10	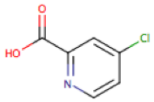

Supplementary Fig. 3

A

Name	Structure
239852	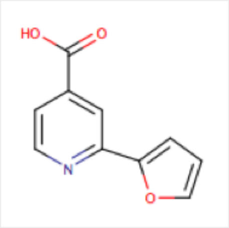
00804	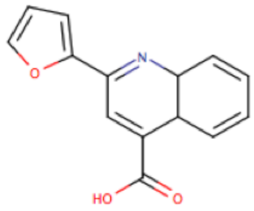
25321	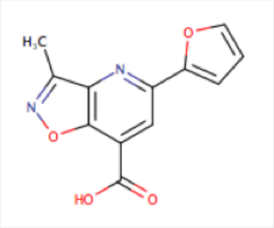
86893	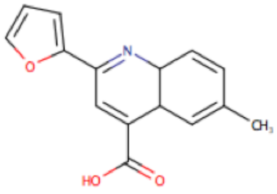
105055	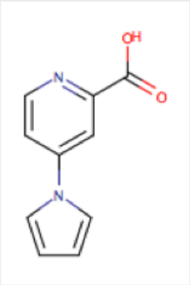

B

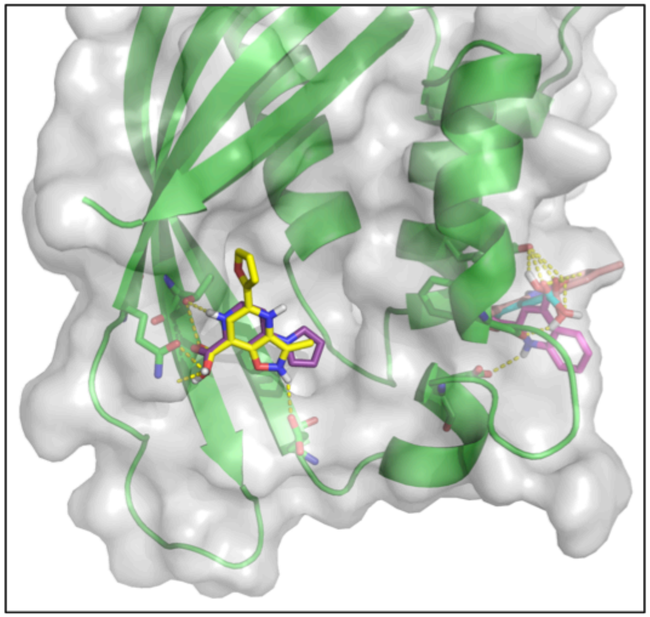

C

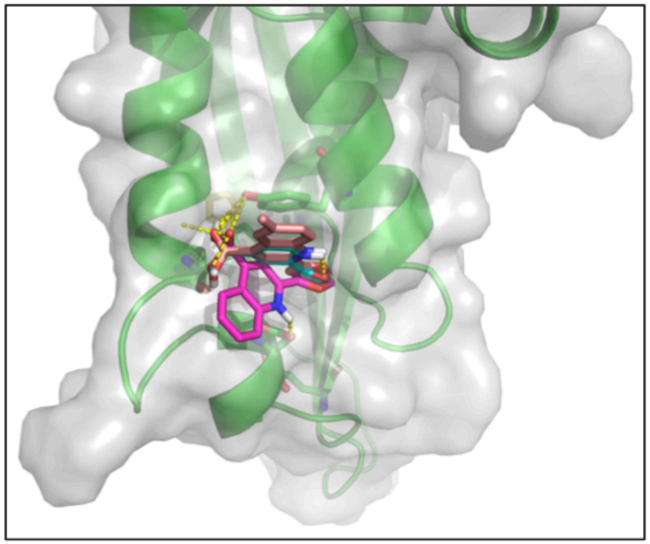

Supplementary Fig. 4

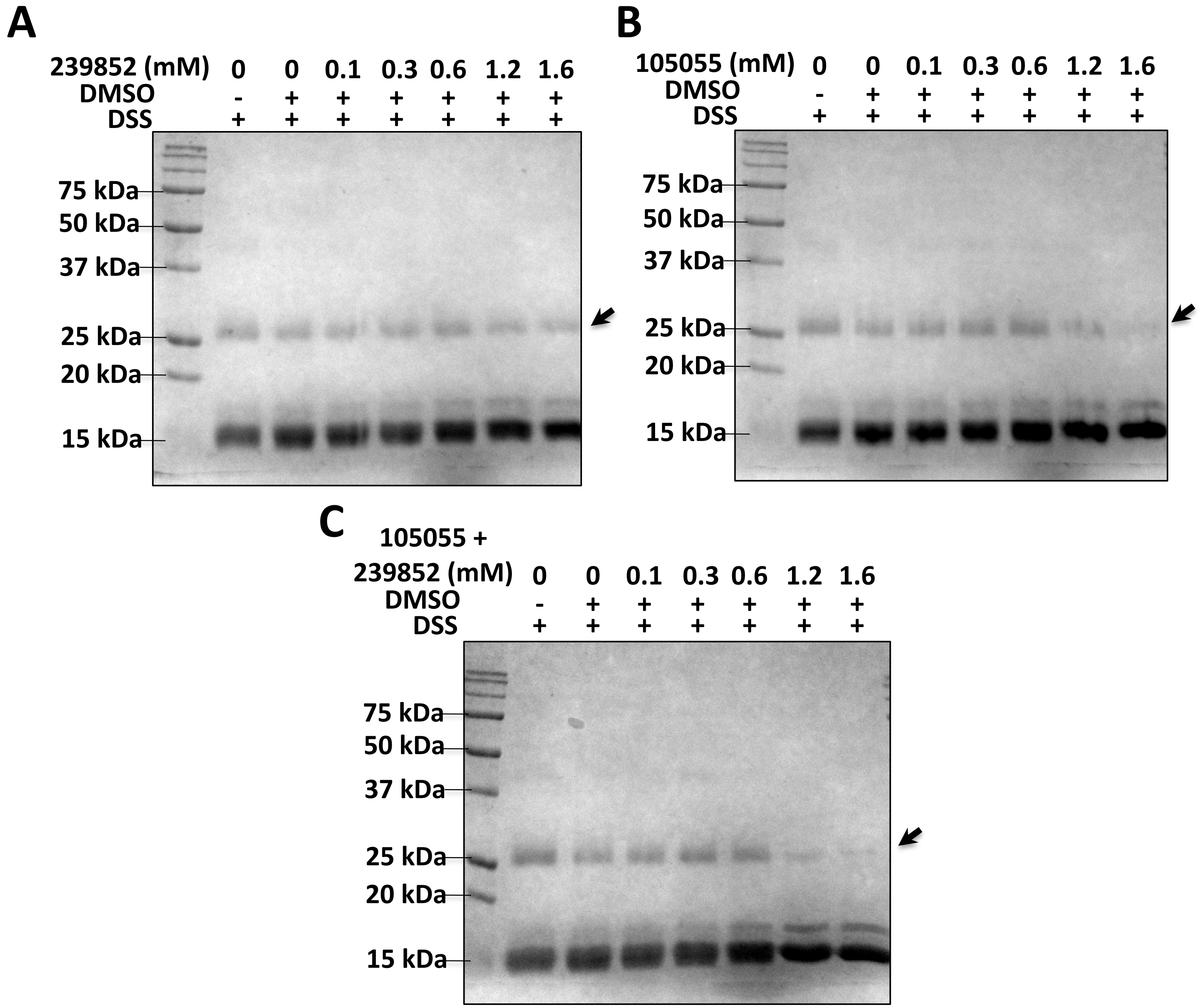

Supplementary Fig. 5

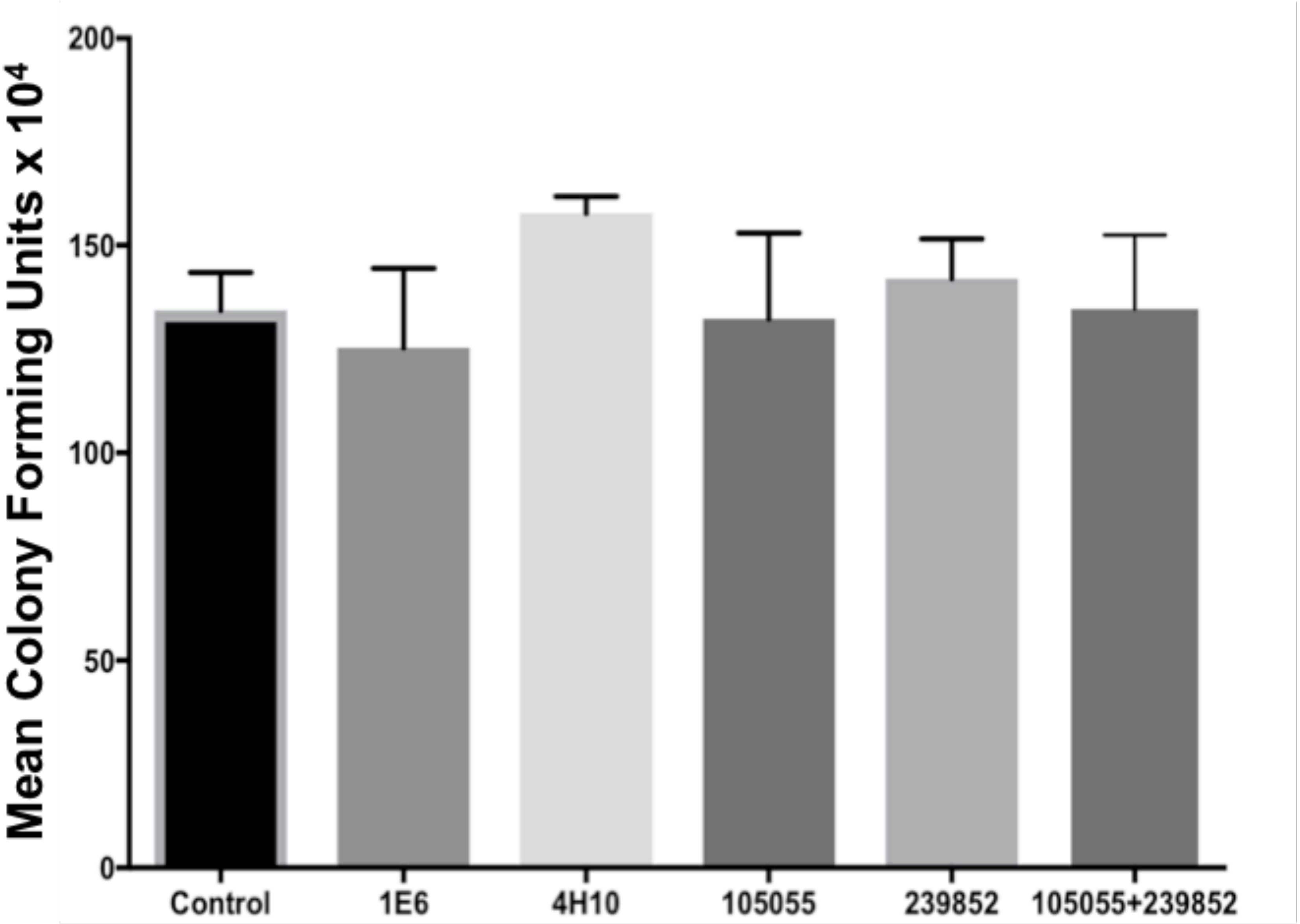

	Structure	Name	Molecular weight (Da)
1	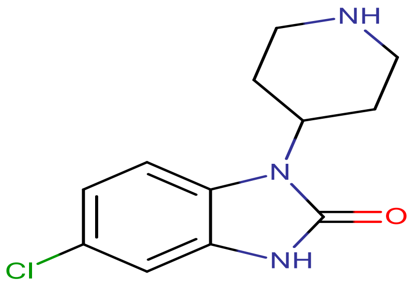	5-CHLORO-1-(4-PIPERIDINYL)-2-BENZIMIDAZOLIDINONE	251.7
2	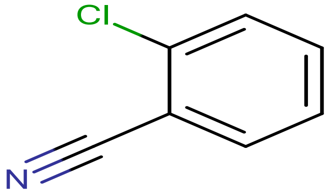	2-CHLOROBENZONITRILE	137.6
3	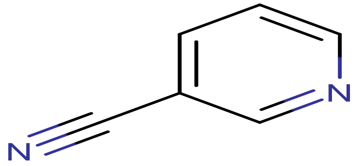	3-CYANOPYRIDINE	104.1
4	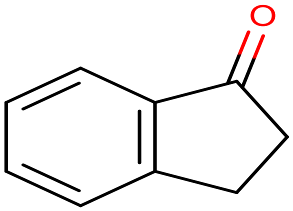	1-INDANONE	132.2

5	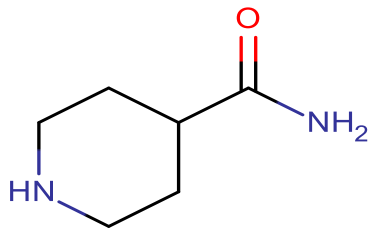	ISONIPECOTAMIDE	128.2
6	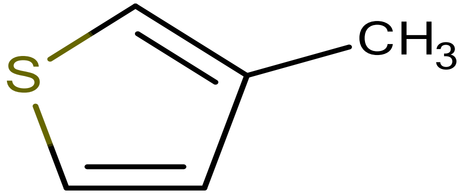	3-METHYLTHIOPHENE	98.2
7	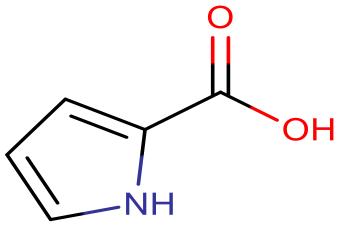	PYRROLE-2-CARBOXYLIC ACID	111.1
8	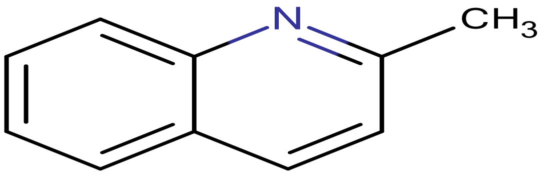	QUINALDINE	143.2

9	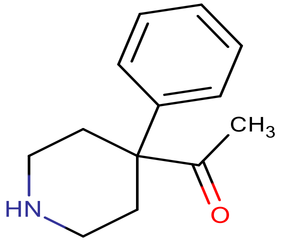	4-ACETYL-4-PHENYLPIPERIDINE HYDROCHLORIDE 99% 25GM	239.7
10	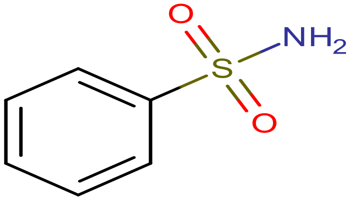	BENZENESULFONAMIDE	157.2
11	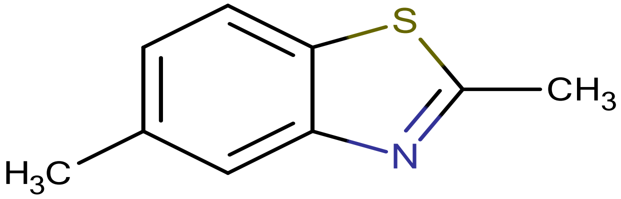	2,5-DIMETHYLBENZOTHIAZOLE	163.2
12	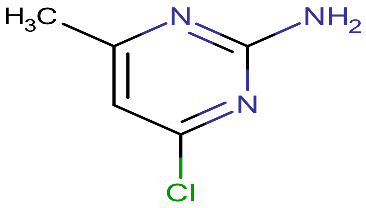	2-AMINO-4-CHLORO-6-METHYLPYRIMIDINE	143.6

13	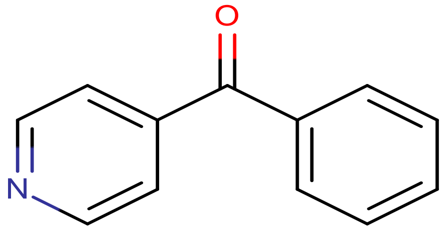	4-BENZOYLPYRIDINE, 98%	183.2
14	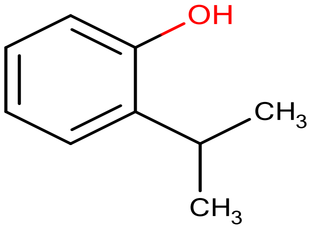	2-ISOPROPYLPHENOL	136.2
15	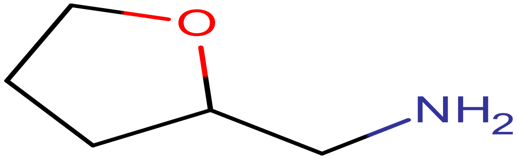	TETRAHYDROFURFURYLAMINE	101.1
16	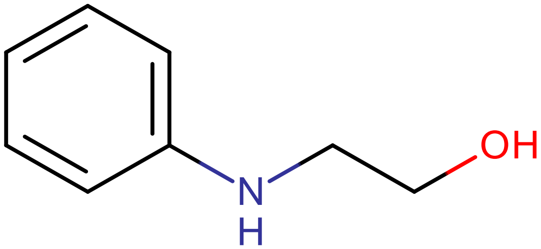	2-ANILINOETHANOL	137.2

17	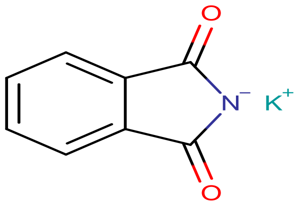	PHTHALIMIDE POTASSIUM DERIVATIVE	185.2
18	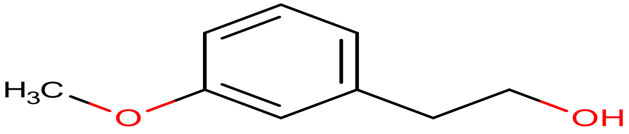	3-METHOXYPHENETHYL ALCOHOL	152.2
19	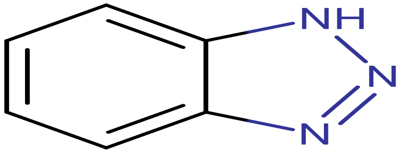	BENZOTRIAZOLE	119.1
20	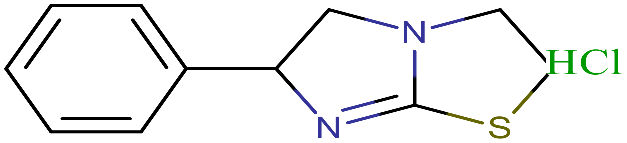	(-)-Tetramisole hydrochloride	240.8
21	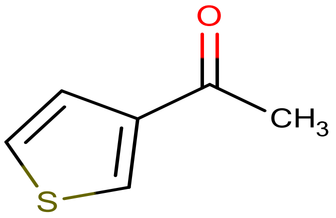	3-ACETYLTHIOPHENE	126.2

22	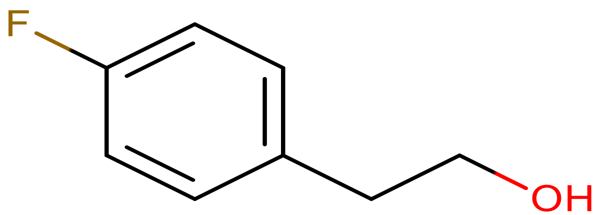	4-FLUOROPHENETHYL ALCOHOL	140.2
23	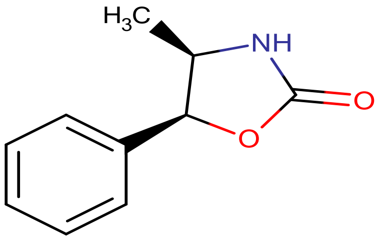	(4R,5S)-(+)-4-METHYL-5-PHENYL-2-OXAZOLIDINONE 99%	177.2
24	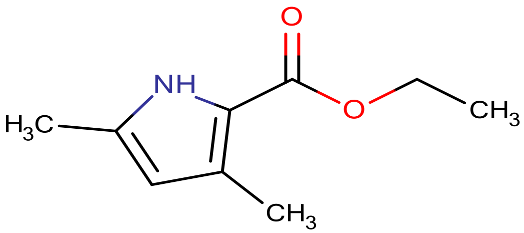	Ethyl 3,5-dimethyl-2-pyrrolecarboxylate	167.2
25	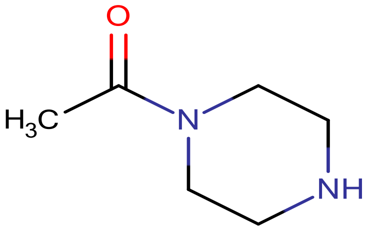	1-ACETYLPIPERAZINE	128.2

26	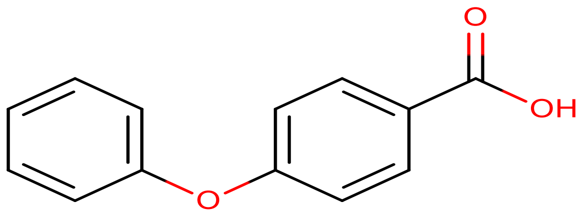	4-Phenoxybenzoic acid	214.2
27	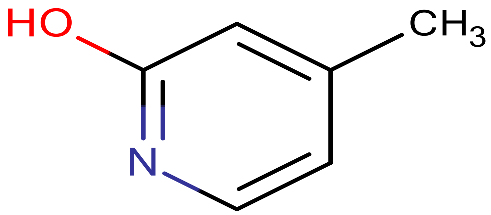	2-HYDROXY-4-METHYLPYRIDINE	109.1
28	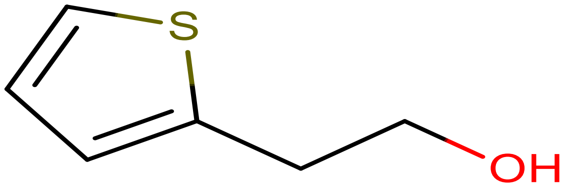	2-(2-THIENYL)ETHANOL	128.2
29	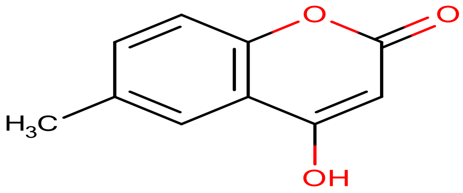	4-Hydroxy-6-methylcoumarin	176.2

30	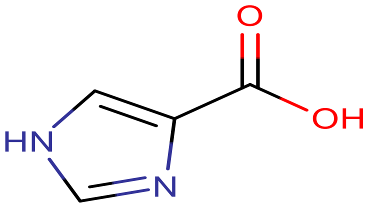	4-IMIDAZOLECARBOXYLIC ACID	112.1
31	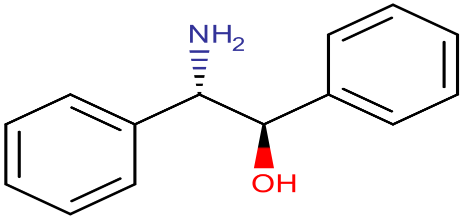	(1R,2S)-(-)-2-AMINO-1,2-DIPHENYLETHANOL	213.3
32	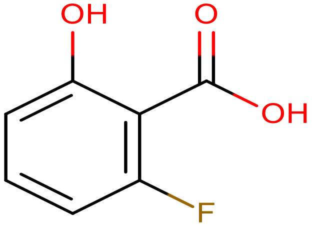	6-FLUORO-2-HYDROXYBENZOIC ACID	156.1
33	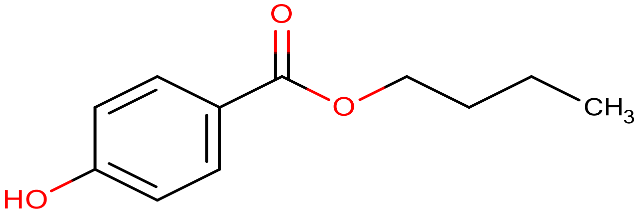	4-HYDROXYBENZOIC ACID BUTYL ESTER	194.2

34	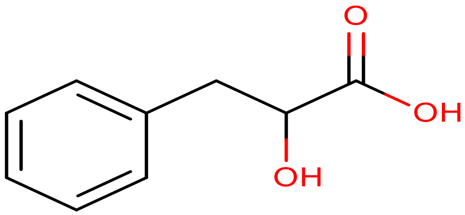	(+/-)-3-Phenyllactic acid	166.2
35	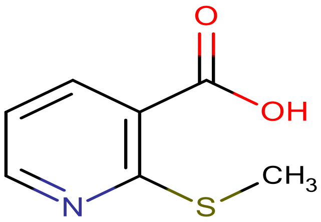	2-(METHYLTHIO)NICOTINIC ACID, 98%	169.2
36	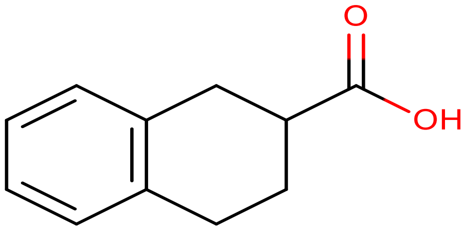	1,2,3,4-TETRAHYDRO-2-NAPHTHOIC ACID	176.2
37	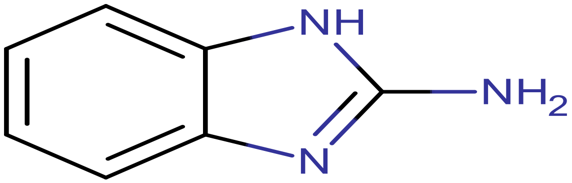	2-aminobenzimidazole	133.2

38	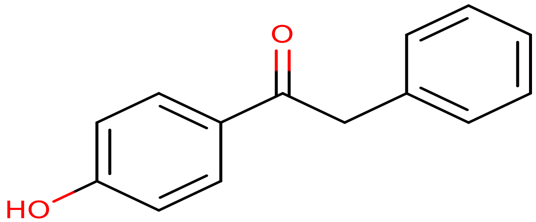	BENZYL 4-HYDROXYPHENYL KETONE	212.2
39	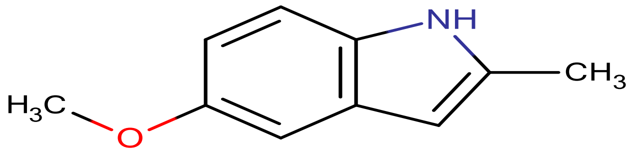	5-METHOXY-2-METHYLINDOLE	161.2
40	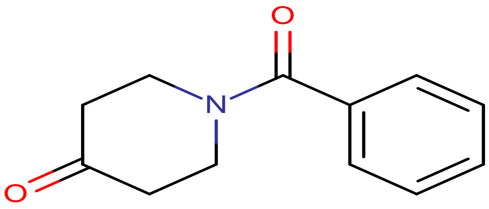	1-Benzoyl-4-piperidone, 97%	203.2
41	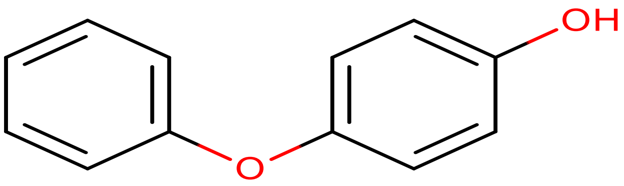	4-Phenoxy phenol	186.2

42	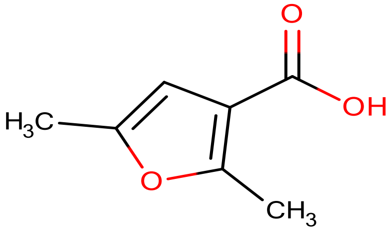	2,5-Dimethyl-3-furoic acid	140.1
43	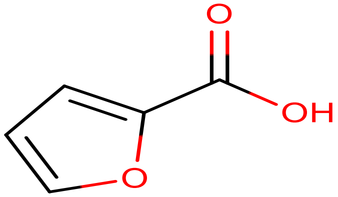	2-FUROIC ACID	112.1
44	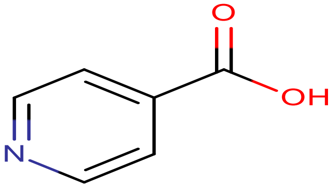	ISONICOTINIC ACID	123.1
45	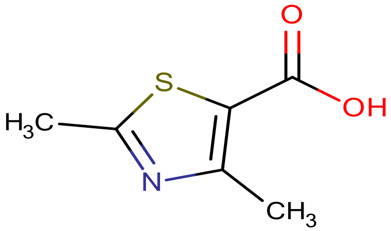	2,4-DIMETHYL-1,3-THIAZOLE-5-CARBOXLIC ACID	157.2

46	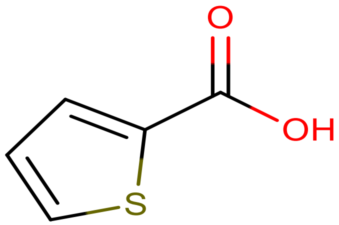	2-THIOPHENECARBOXYLIC ACID	128.2
47	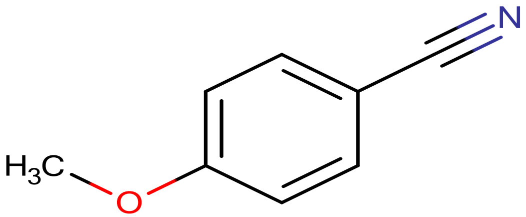	4-METHOXYBENZONITRILE	133.1
48	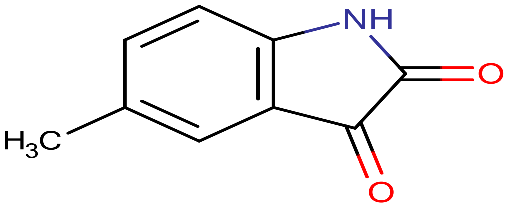	5-METHYLISATIN, 97%	161.2
49	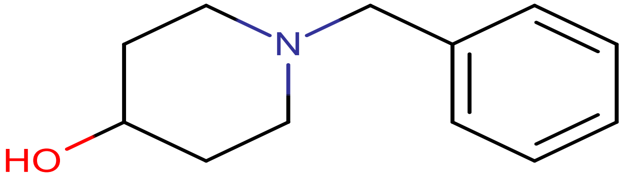	1-BENZYL-4-HYDROXYPIPERIDINE	191.3

50	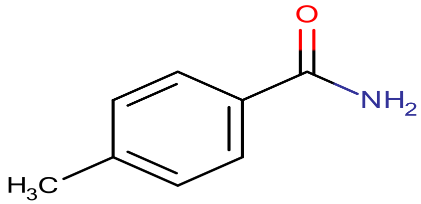	p-Toluamide	135.2
51	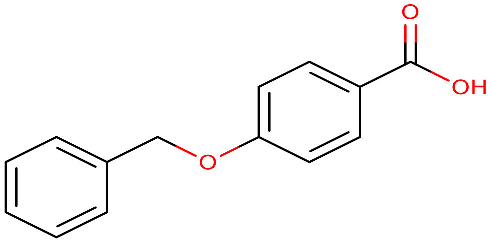	4-BENZYLOXYBENZOIC ACID	228.2
52	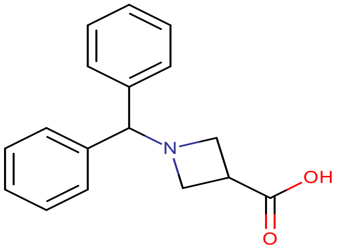	1-BENZHYDRYLAZETANE-3-CARBOXYLIC ACID	267.3
53	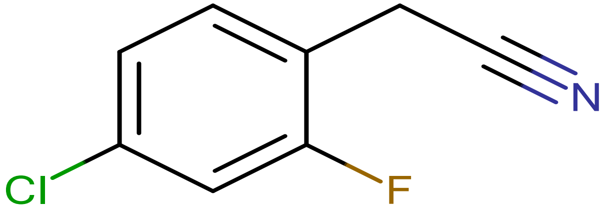	2-(4-Chloro-2-fluorophenyl)acetonitrile	169.6

54	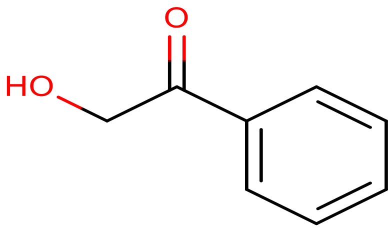	2-HYDROXYACETOPHENONE	136.1
55	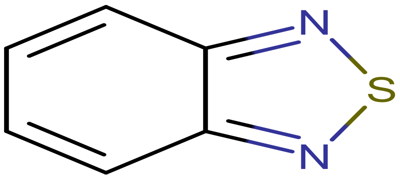	2,1,3-BENZOTHIADIAZOLE	136.2
56	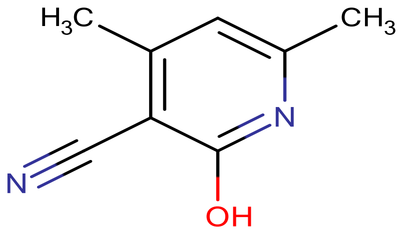	2-hydroxy-4,6-dimethylpyridine-3-carbonitrile	148.2
57	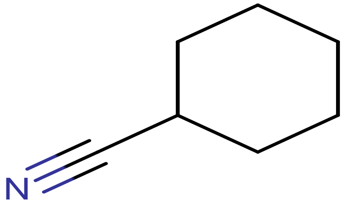	CYCLOHEXANECARBONITRILE	109.2

58	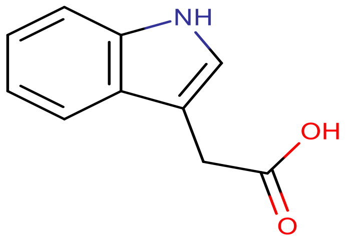	INDOLE-3-ACETIC ACID	175.2
59	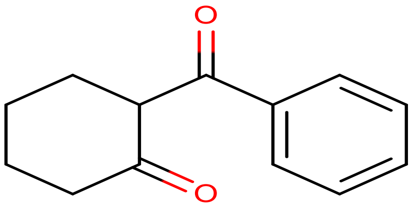	2-BENZOYLCYCLOHEXANONE	202.3
60	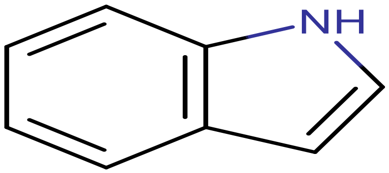	INDOLE	117.2
61	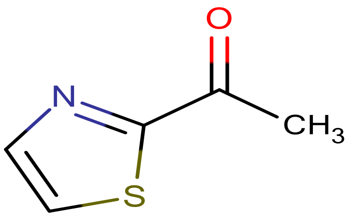	2-Acetylthiazole	127.2

62	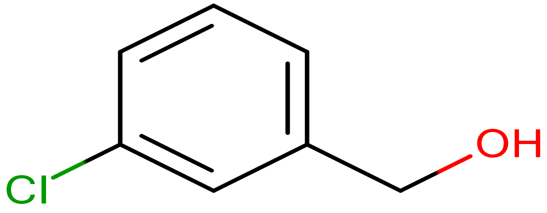	3-CHLOROBENZYL ALCOHOL	142.6
63	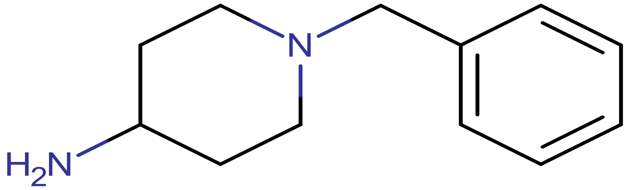	4-amino-1-benzylpiperidine,98%	190.3
64	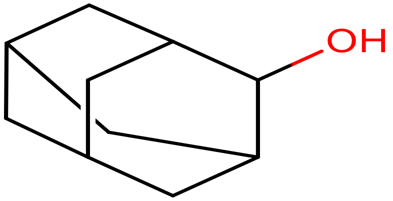	2-ADAMANTANOL	152.2
65	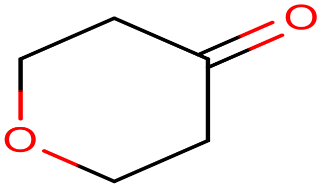	TETRAHYDRO-4H-PYRAN-4-ONE	100.1

66	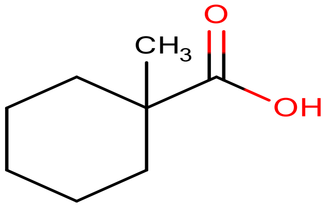	1-METHYL-1-CYCLOHEXANECARBOXYLIC ACID, 99%	142.2
67	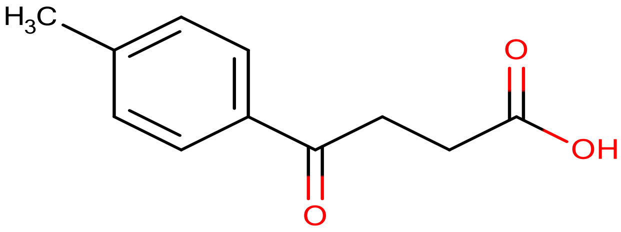	3-(4-METHYLBENZOYL)PROPIONIC ACID	192.2
68	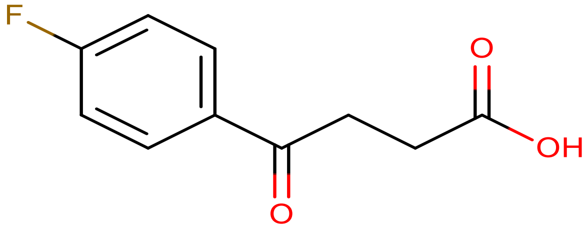	3-(4-FLUOROBENZOYL)PROPIONIC ACID	196.2
69	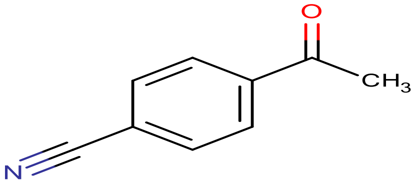	4-ACETYLBENZONITRILE	145.2
70	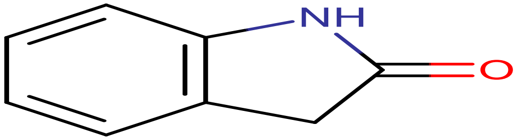	Oxindole	133.1

71	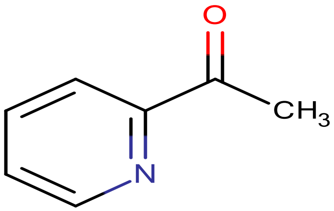	2-ACETYLPYRIDINE	121.1
72	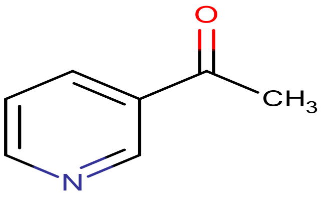	3-ACETYLPYRIDINE	121.1
73	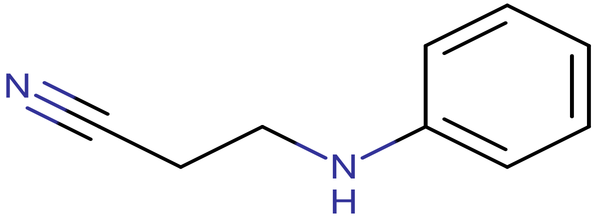	3-ANILINOPROPIONITRILE	146.2
74	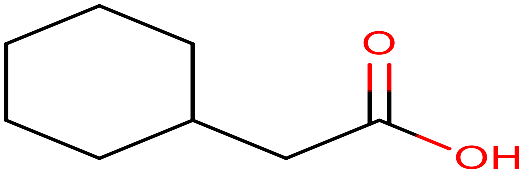	CYCLOHEXYLACETIC ACID	142.2

75	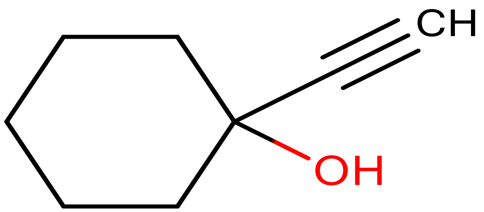	1-ETHYNYL-1-CYCLOHEXANOL	124.2
76	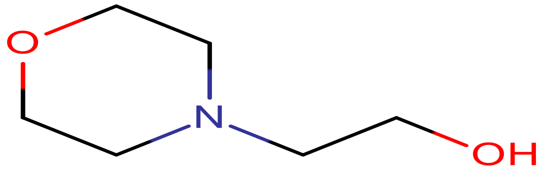	4-(2-HYDROXYETHYL)MORPHOLINE	131.2
77	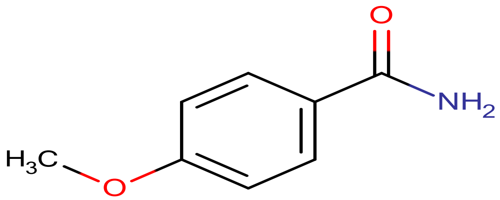	4-METHOXYBENZAMIDE	151.2
78	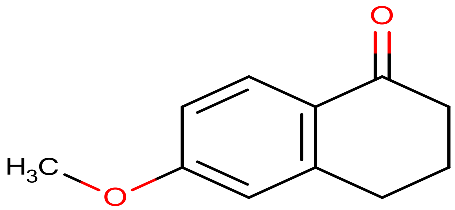	6-METHOXY-1-TETRALONE	176.2

79	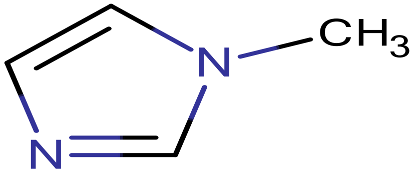	1-Methylimidazole	82.1
80	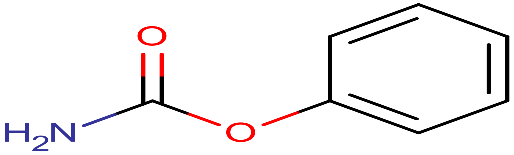	PHENYL CARBAMATE	137.1
81	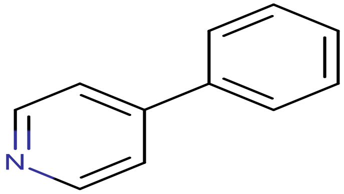	4-PHENYLPYRIDINE	155.2
82	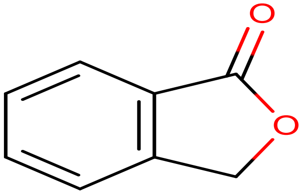	PHTHALIDE	134.1
83	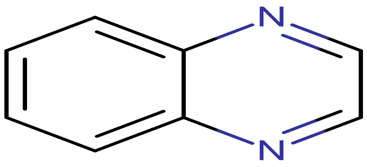	QUINOXALINE	130.1

84	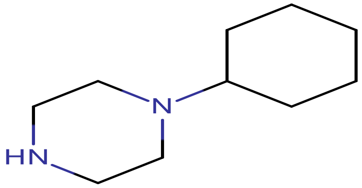	1-CYCLOHEXYLPIPERAZINE	168.3
85	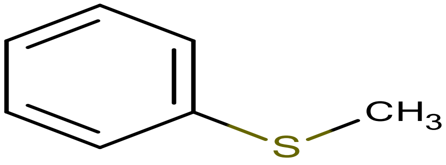	Thioanisole	124.2
86	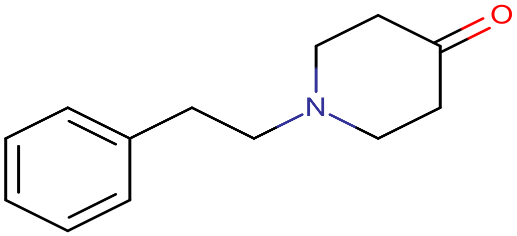	1-(B-PHENETHYL)-4-PIPERIDONE	203.3
87	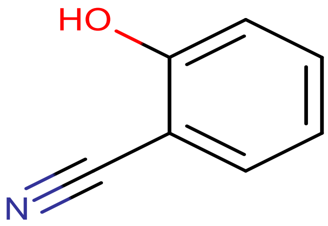	2-CYANOPHENOL	119.1

88	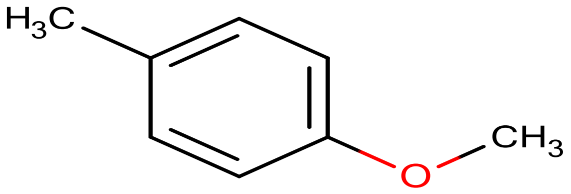	4-METHYLANISOLE	122.2
89	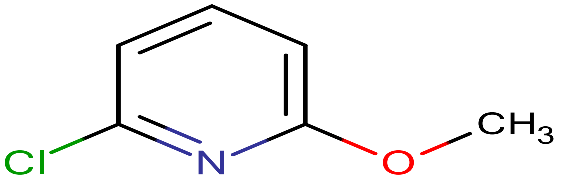	2-CHLORO-6-METHOXYPYRIDINE	143.6
90	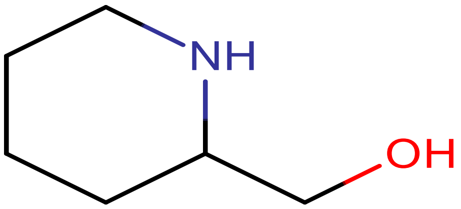	2-Piperidinemethanol tech., 93%	115.2
91	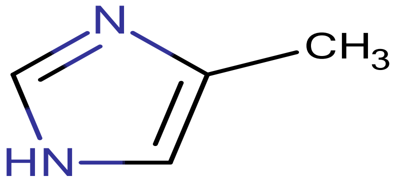	4-METHYLIMIDAZOLE	82.1
92	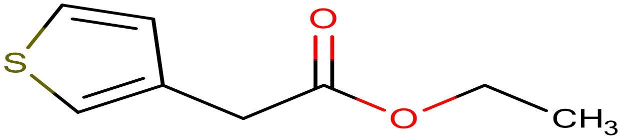	ETHYL 3-THIOPHENEACETATE	170.2

93	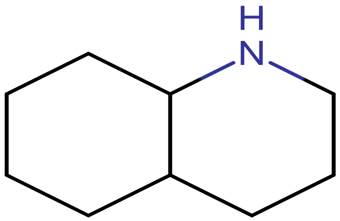	DECAHYDROQUINOLINE, 97%, MIXTURE OF CIS AND TRANS	139.2
94	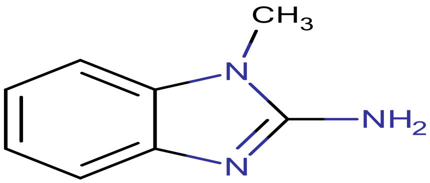	2-AMINO-1-METHYLBENZIMIDAZOLE	147.2
95	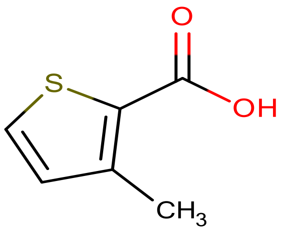	3-METHYL-2-THIOPHENECARBOXYLIC ACID	142.2
96	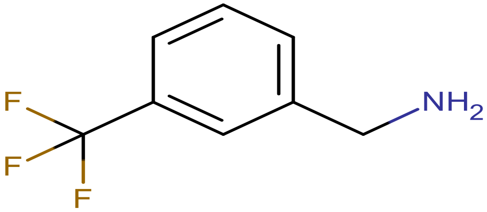	3-(Trifluoromethyl)benzylamine,98%	175.2

97	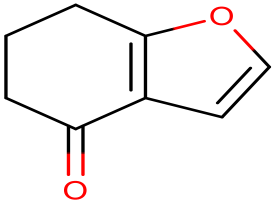	6,7-Dihydro-4(5H)-benzofuranone	136.1
98	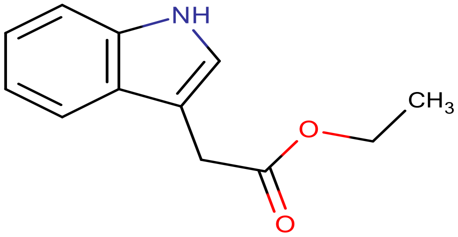	ETHYL 3-INDOLEACETATE	203.2
99	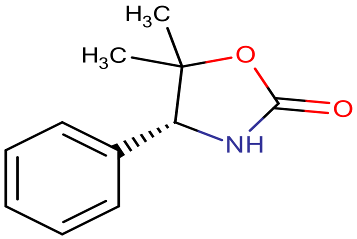	(R)-(-)-5,5-DIMETHYL-4-PHENYL-2-OXAZOLIDINONE	191.2
100	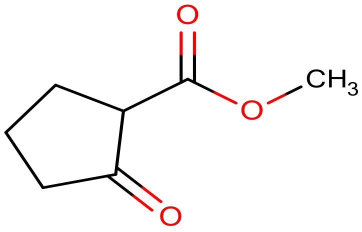	METHYL2-OXOCYCLOPENTANECARBOXYLATE	142.2

101	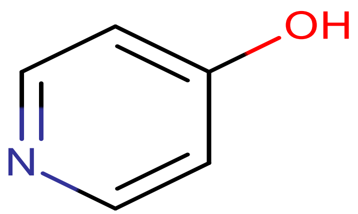	4-HYDROXYPYRIDINE	95.1
102	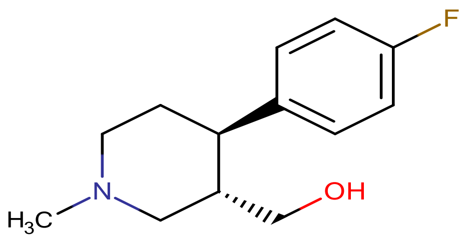	(3S,4R)-4-(4-FLUOROPHENYL)-1-METHYL-3-PIPERIDINEMETHANOL	223.3
103	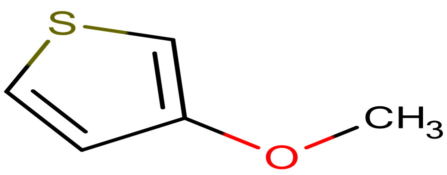	3-Methoxythiophene,98%	114.2
104	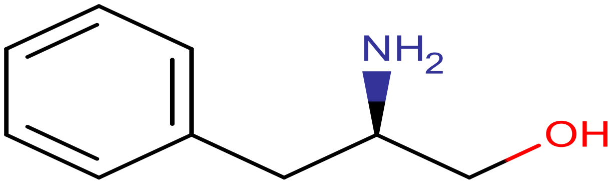	(R)-(+)-2-AMINO-3-PHENYL-1-PROPANOL	151.2

105	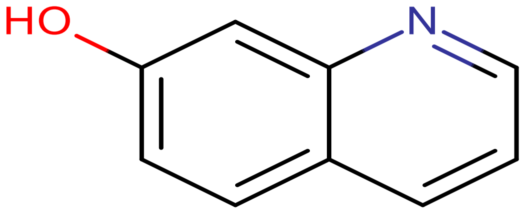	7-hydroxyquinoline	145.2
106	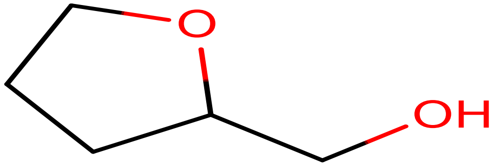	TETRAHYDROFURFURYL ALCOHOL	102.1
107	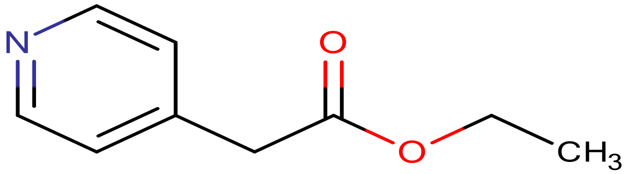	ETHYL 4-PYRIDYLACETATE	165.2
108	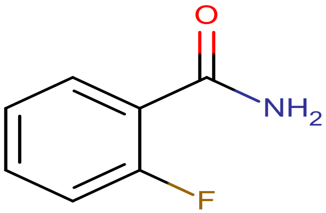	2-FLUOROBENZAMIDE	139.1

109	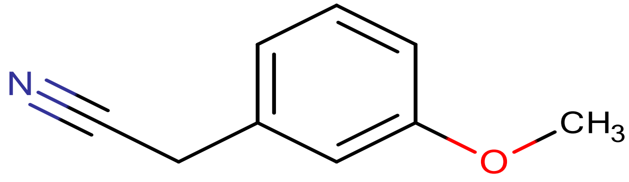	(3-METHOXYPHENYL)ACETONITRILE	147.2
110	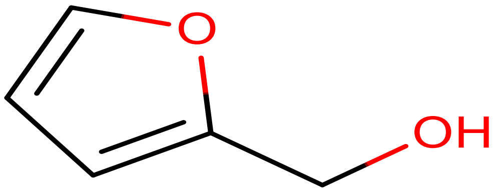	FURFURYL ALCOHOL	98.1
111	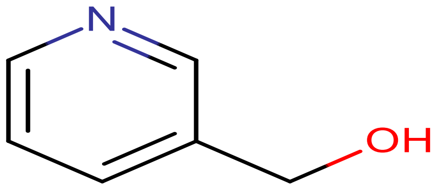	3-PYRIDYLCARBINOL	109.1
112	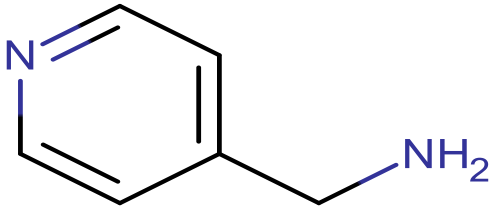	4-(AMINOMETHYL)PYRIDINE	108.1

113	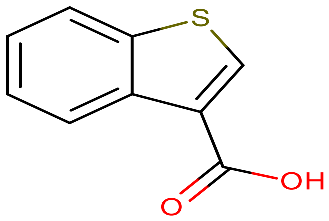	1-BENZOTHIOPHENE-3-CARBOXYLIC ACID, 97%	178.2
114	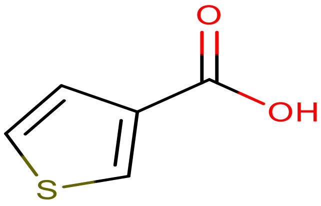	3-THIOPHENECARBOXYLIC ACID	128.2
115	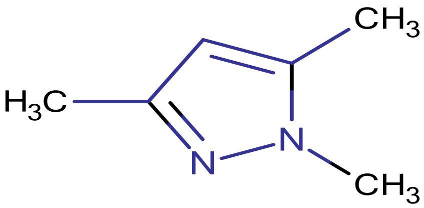	1,3,5-TRIMETHYLPYRAZOLE	110.2
116	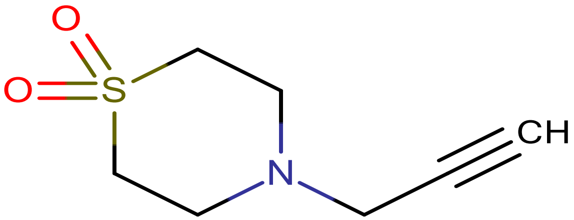	4-PROP-2-YNYL-1LAMBDA6,4-THIAZINANE-1,1-DIONE	173.2

117	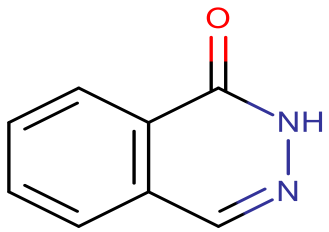	1-(2H)-PHTHALAZINONE	146.1
118	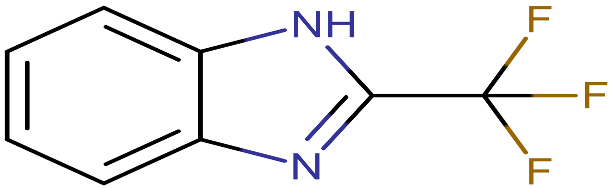	2-(TRIFLUOROMETHYL)BENZIMIDAZOLE	186.1
119	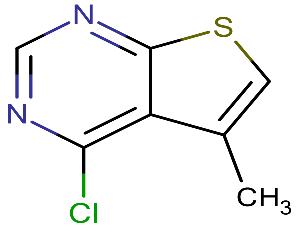	4-Chloro-5-methylthieno[2,3-d]pyrimidine	184.6
120	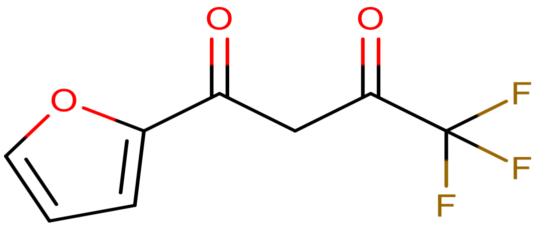	4,4,4-TRIFLUORO-1-(2-FURYL)-1,3-BUTANEDIONE	206.1

121	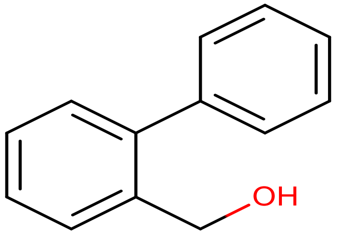	2-BIPHENYLMETHANOL	184.2
122	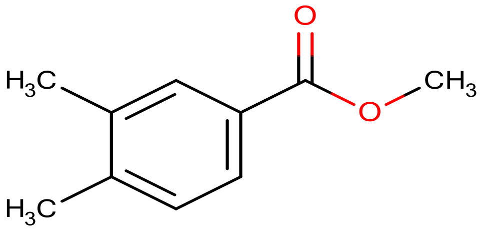	METHYL 3,4-DIMETHYLBENZOATE	164.2
123	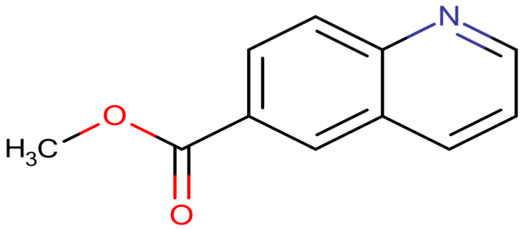	METHYL QUINOLINE-6-CARBOXYLATE	187.2
124	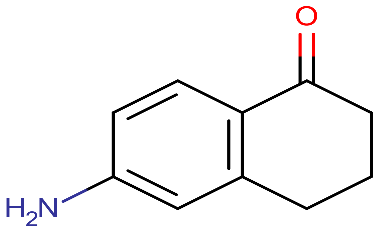	6-AMINO-1,2,3,4-TETRAHYDRONAPHTHALENE-1-ONE	161.2

125	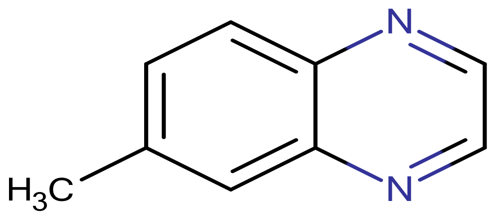	6-METHYLQUINOXALINE	144.2
126	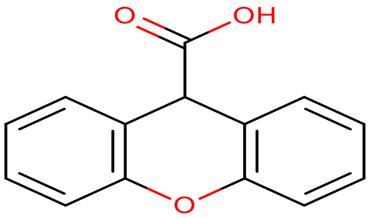	XANTHENE-9-CARBOXYLIC ACID	226.2
127	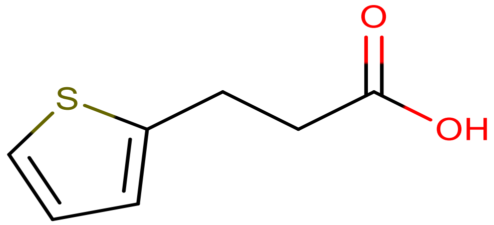	3-(2-THIENYL)PROPANOIC ACID 98%	156.2
128	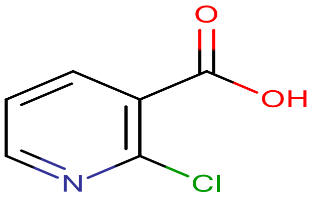	2-CHLORONICOTINIC ACID	157.6

129	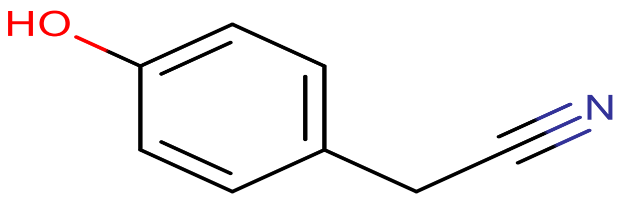	4-Hydroxybenzyl Cyanide	133.1
130	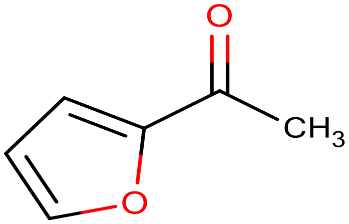	2-ACETYLFURAN	110.1
131	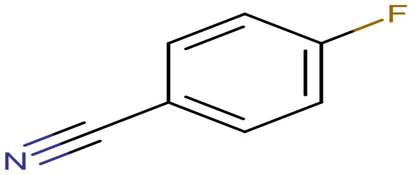	4-FLUOROBENZONITRILE	121.1
132	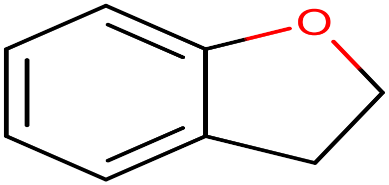	2,3-DIHYDROBENZOFURAN	120.2

133	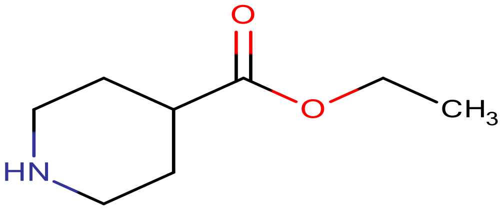	ETHYL ISONIPECOTATE	157.2
134	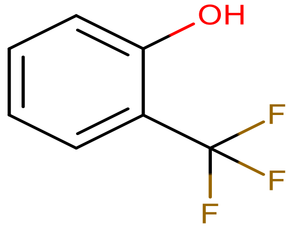	2-(TRIFLUOROMETHYL)PHENOL, 97%	162.1
135	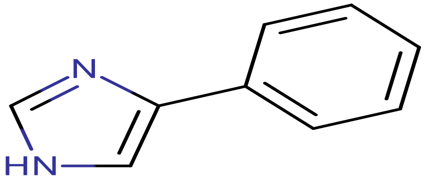	4-PHENYLIMIDAZOLE	144.2
136	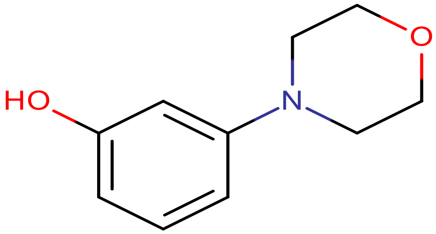	3-(4-morpholino)phenol	179.2

137	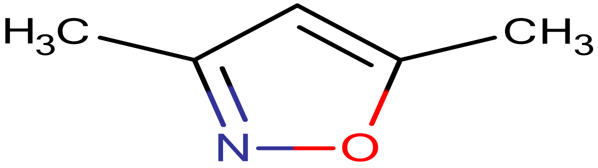	3,5-DIMETHYLISOXAZOLE	97.1
138	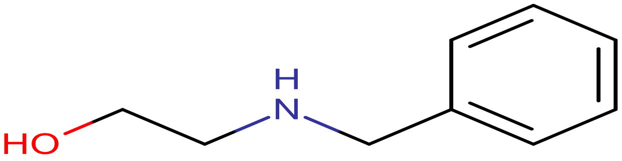	N-BENZYLETHANOLAMINE	151.2
139	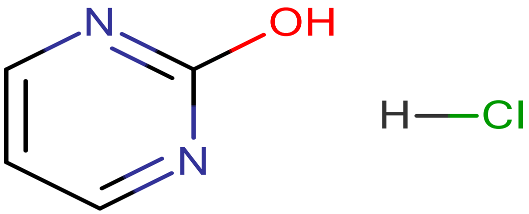	2-HYDROXYPYRIMIDINE HYDROCHLORIDE	132.5
140	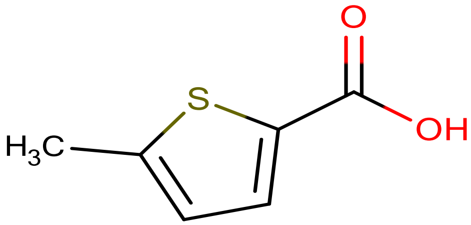	5-METHYL-2-THIOPHENECARBOXYLIC ACID	142.2

141	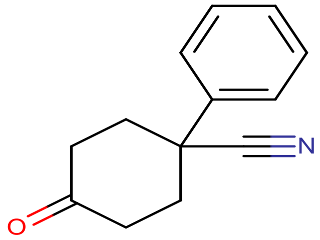	4-CYANO-4-PHENYLCYCLOHEXANONE	199.3
142	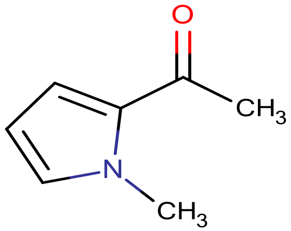	2-ACETYL-1-METHYLPYRROLE	123.2
143	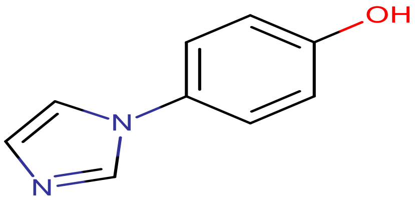	4-(IMIDAZOL-1-YL)PHENOL	160.2
144	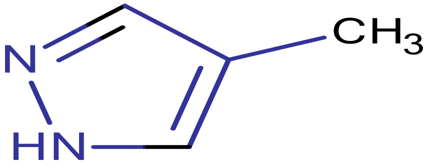	4-METHYLPYRAZOLE	82.1

145	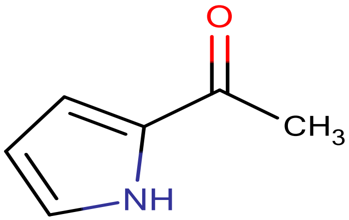	2-ACETYLPYRROLE	109.1
146	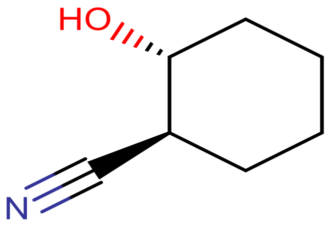	TRANS-2-CYANO-1-CYCLOHEXANOL	125.2
147	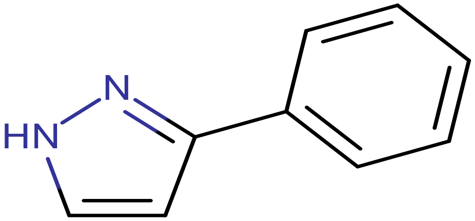	3-PHENYL-1H-PYRAZOLE	144.2

148	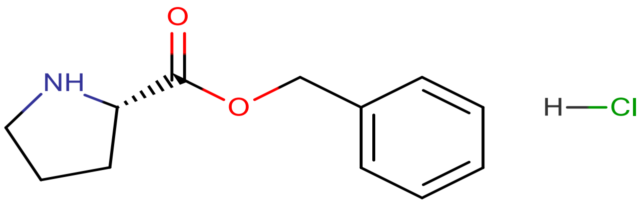	L-PROLINE BENZYL ESTER HYDROCHLORIDE	241.7
149	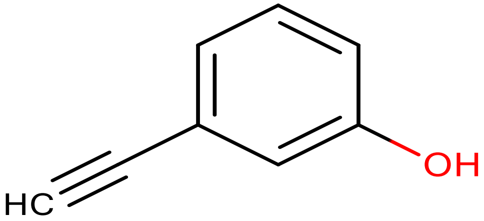	3-HYDROXYPHENYLACETYLENE, TECH.	118.1
150	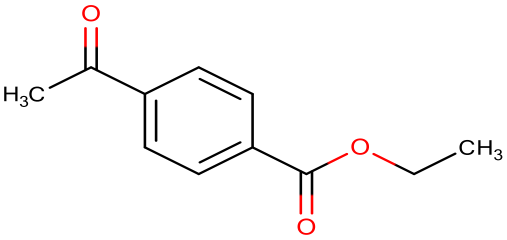	ETHYL 4-ACETYLBENZOATE	192.2

151	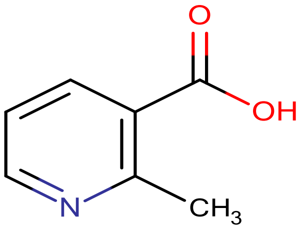	2-METHYLNICOTINIC ACID	137.1
152	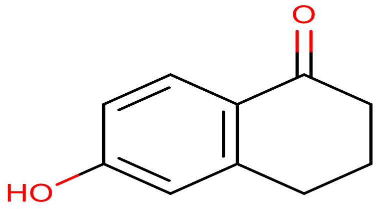	6-HYDROXY-1-TETRALONE	162.2
153	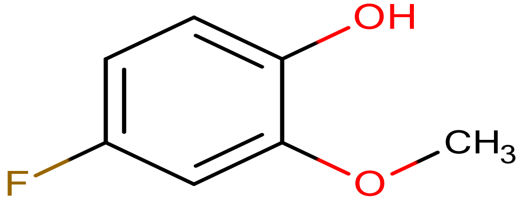	4-Fluoro-2-methoxyphenol	142.1
154	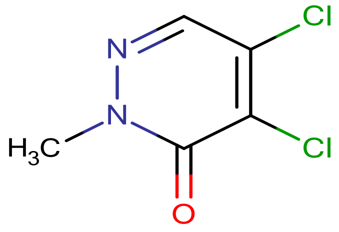	4,5-DICHLORO-2-METHYLPYRIDAZIN-3(2H)-ONE	179.0

155	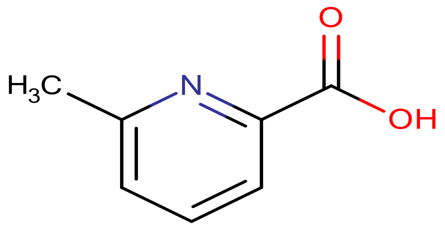	6-METHYLPICOLINIC ACID	137.1
156	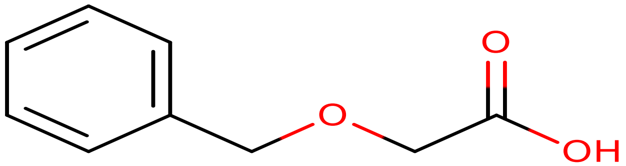	BENZYLOXYACETIC ACID	166.2
157	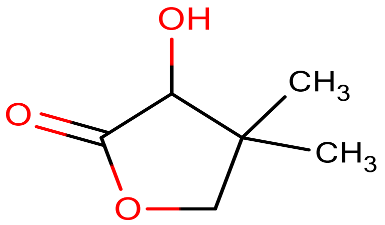	(+/-)-PANTOLACTONE, 99%	130.1
158	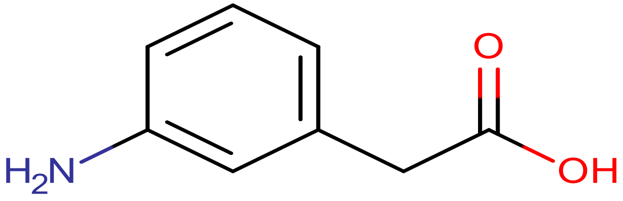	(3-AMINOPHENYL)ACETIC ACID	151.2

159	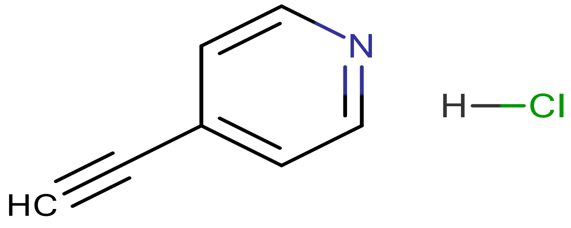	4-ETHYNYLPYRIDINE HYDROCHLORIDE	139.6
160	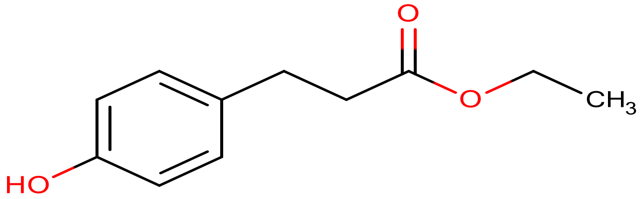	ETHYL 3-(4-HYDROXYPHENYL)PROPANOATE	194.2
161	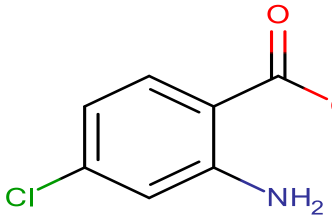	2-AMINO-4-CHLOROBENZOIC ACID	171.6
162	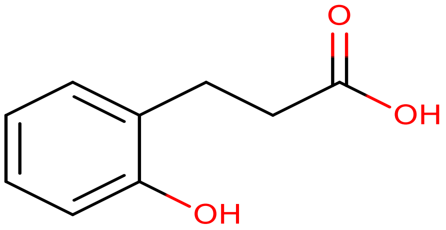	3-(2-Hydroxyphenyl)propionic acid	166.2

163	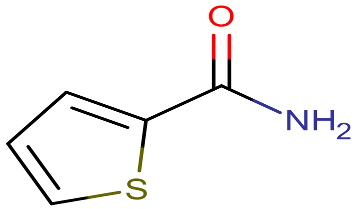	2-Thiophenecarboxamide	127.2
164	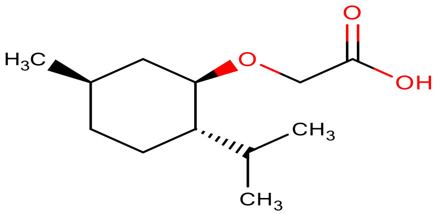	L-MENTHOXYACETIC ACID	214.3
165	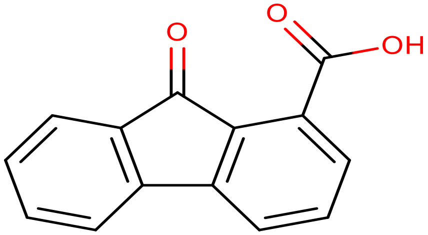	9-FLUORENONE-1-CARBOXYLIC ACID	224.2
166	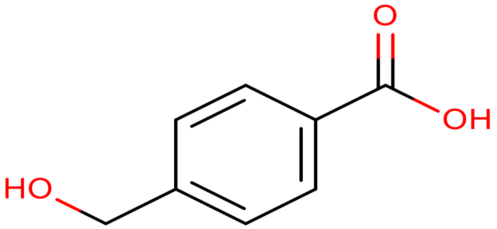	4-(HYDROXYMETHYL)BENZOIC ACID	152.1

167	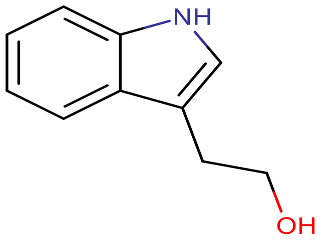	TRYPTOPHOL	161.2
168	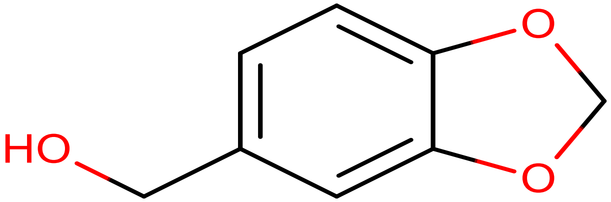	Piperonyl alcohol	152.1
169	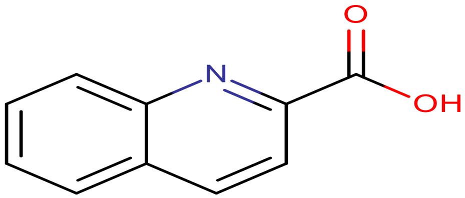	QUINALDIC ACID	173.2
170	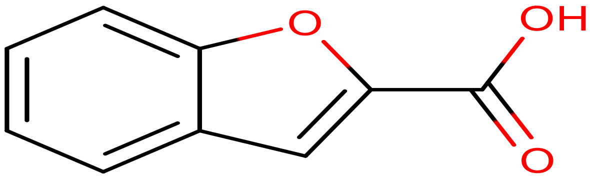	2-Benzofurancarboxylic acid	162.1

171	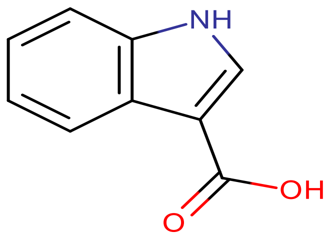	INDOLE-3-CARBOXYLIC ACID	161.2
172	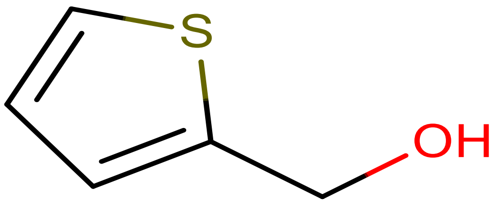	2-THIOPHENEMETHANOL	114.2
173	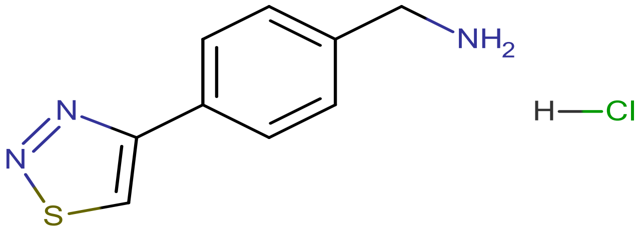	4-(1,2,3-THIADIAZOL-4-YL)BENZYLAMINE HYDROCHLORIDE	227.7
174	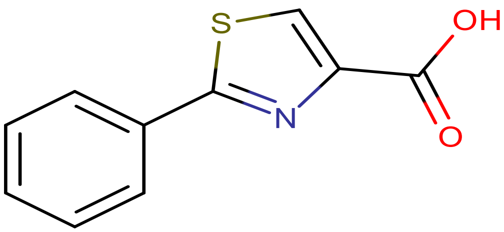	2-PHENYL-1,3-THIAZOLE-4-CARBOXYLIC ACID	205.2

175	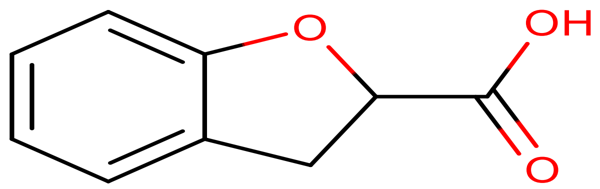	2,3-DIHYDRO-1-BENZOFURAN-2-CARBOXYLIC ACID	164.2
176	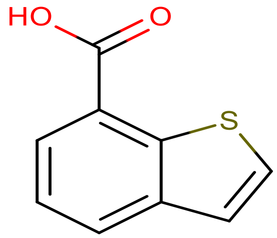	BENZO[B]THIOPHENE-7-CARBOXYLIC ACID	178.2
177	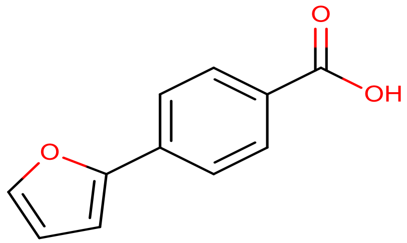	4-(2-FURYL)-BENZOIC ACID	188.2
178	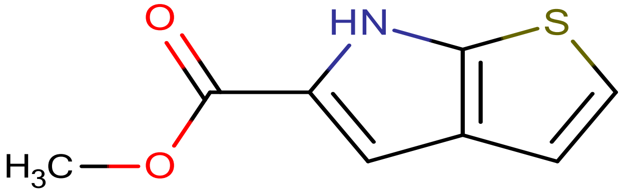	METHYL 6H-THIENO[2,3-B]PYRROLE-5-CARBOXYLATE	181.2

179	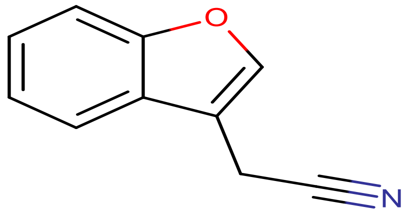	3-benzo[b]furylacetonitrile	157.2
180	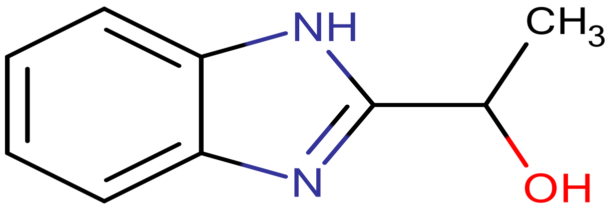	2-(1-HYDROXYETHYL)BENZIMIDAZOLE	162.2
181	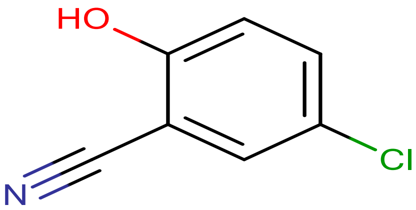	5-CHLORO-2-HYDROXYBENZONITRILE	153.6
182	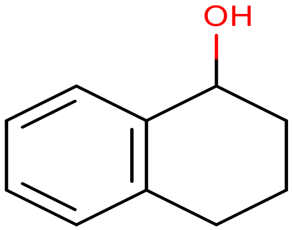	1,2,3,4-TETRAHYDRO-1-NAPHTHOL	148.2

183	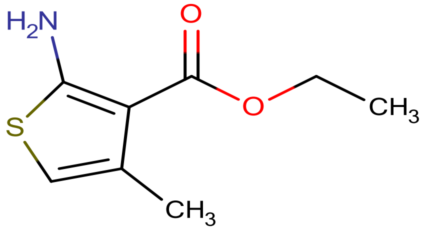	ETHYL 2-AMINO-4-METHYLTHIOPHENE-3-CARBOXYLATE	185.2
184	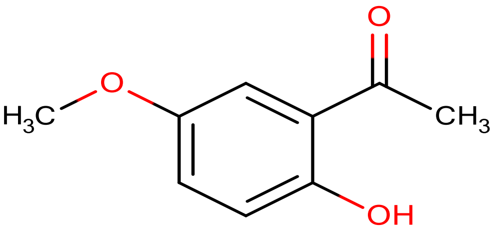	2'-HYDROXY-5'-METHOXYACETOPHENONE	166.2
185	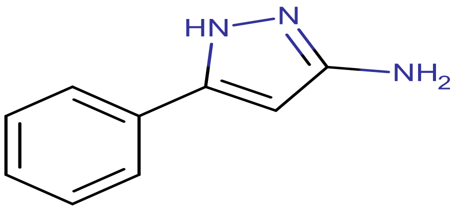	3-AMINO-5-PHENYLPYRAZOLE	159.2
186	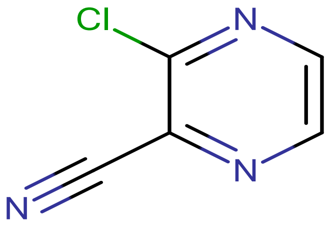	3-chloro pyrazine-2-carbonitrile	139.5

187	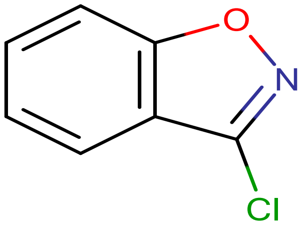	3-Chloro-1,2-benzisoxazole	153.6
188	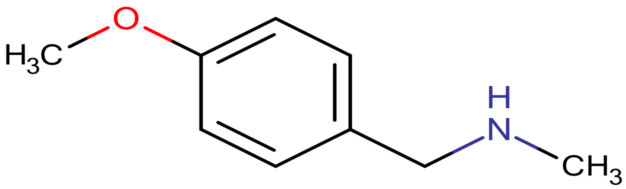	N-(4-METHOXYBENZYL)-N-METHYLAMINE	151.2
189	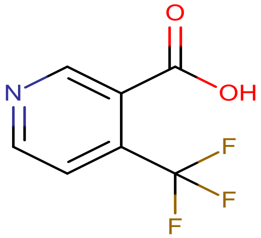	4-(TRIFLUOROMETHYL)NICOTINIC ACID	191.1
190	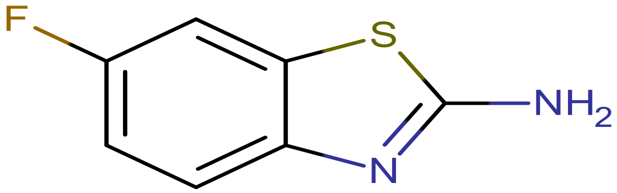	2-amino-6-fluorobenzothiazole	168.2

191	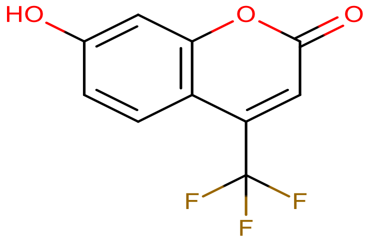	7-HYDROXY-4-(TRIFLUOROMETHYL)-COUMARIN	230.1
192	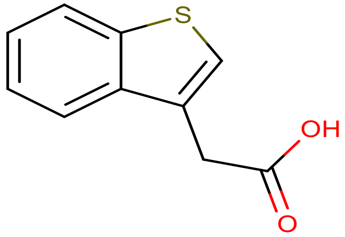	BENZO[B]THIOPHENE-3-ACETIC ACID	192.2
193	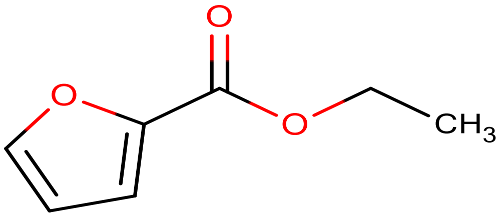	ETHYL 2-FUROATE	140.1
194	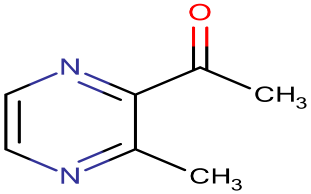	2-ACETYL-3-METHYLPYRAZINE	136.2

195	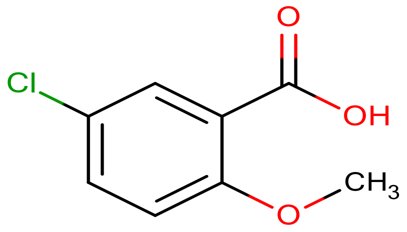	5-CHLORO-2-METHOXYBENZOIC ACID	186.6
196	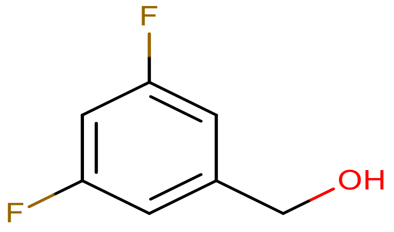	3,5-Difluorobenzyl alcohol,97%	144.1
197	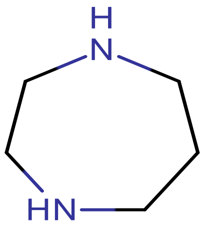	Homopiperazine	100.2
198	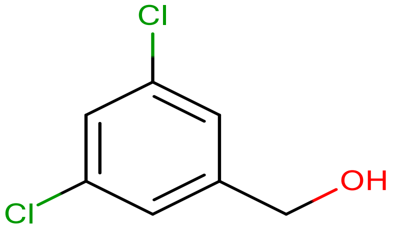	3,5-DICHLOROBENZYL ALCOHOL	177.0

199	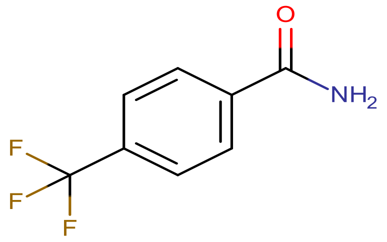	4-(TRIFLUOROMETHYL)BENZAMIDE	189.1
200	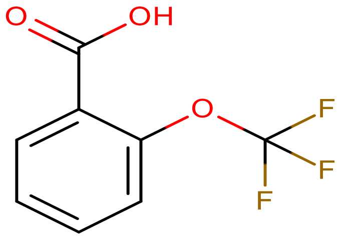	2-(TRIFLUOROMETHOXY)BENZOIC ACID	206.1
201	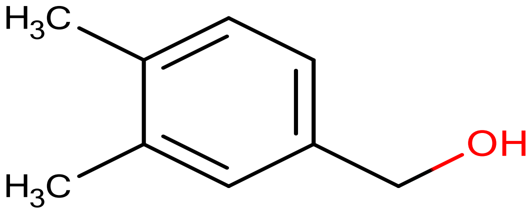	3,4-Dimethylbenzyl alcohol	136.2
202	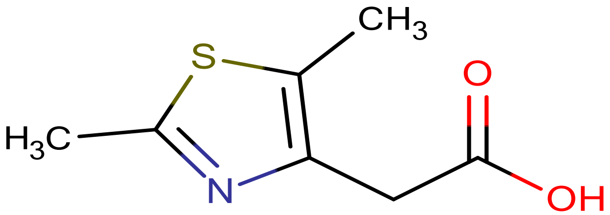	2-(2,5-DIMETHYL-1,3-THIAZOL-4-YL)ACETIC ACID	171.2

203	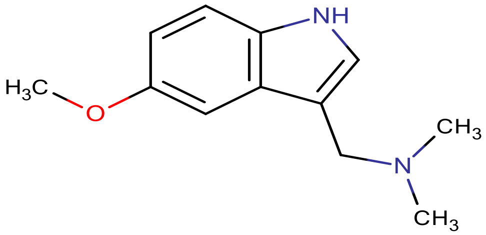	5-METHOXYGRAMINE	204.3
204	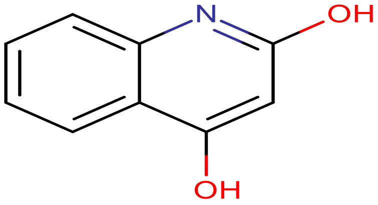	2,4-QUINOLINEDIOL	161.2
205	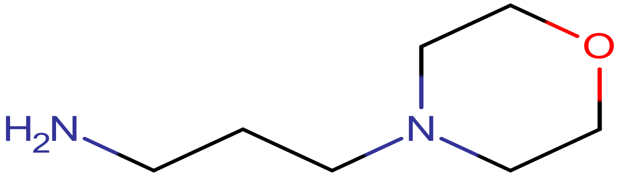	4-(3-AMINOPROPYL)MORPHOLINE	144.2
206	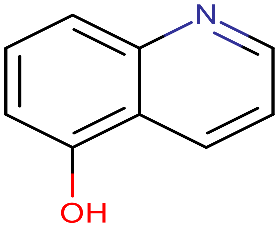	5-hydroxyquinoline	145.2

207	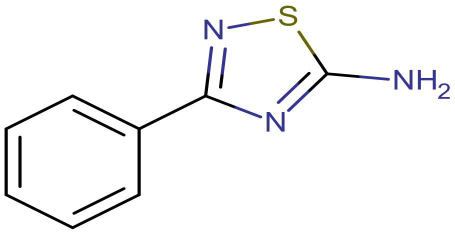	5-AMINO-3-PHENYL-1,2,4-THIADIAZOLE	177.2
208	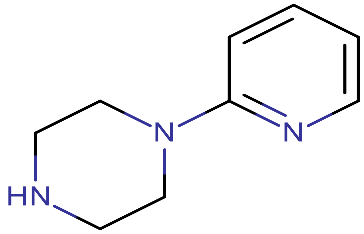	1-(2-PYRIDYL)PIPERAZINE	163.2
209	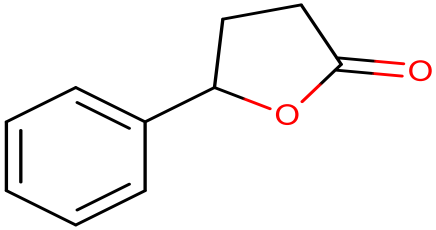	Gamma-Phenyl-Gamma-butyrolactone	162.2
210	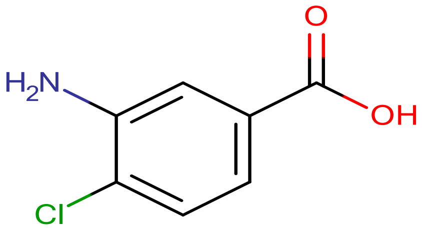	3-AMINO-4-CHLOROBENZOIC ACID	171.6

211	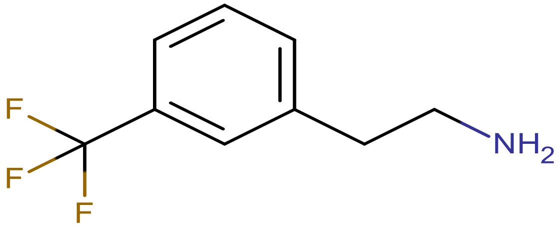	3-(TRIFLUOROMETHYL)PHENETHYLAMINE	189.2
212	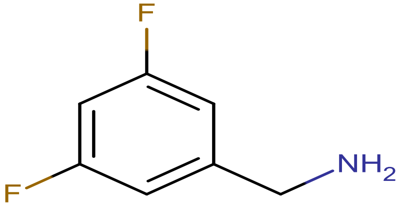	3,5-DIFLUOROBENZYLAMINE, 97%	143.1
213	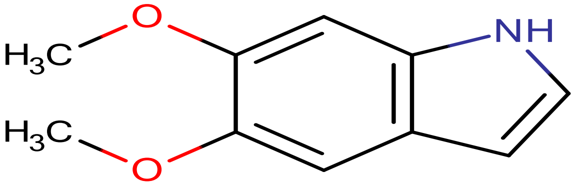	5,6-dimethoxyindole	177.2
214	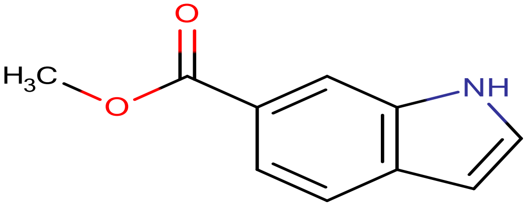	Methyl indole-6-carboxylate	175.2

215	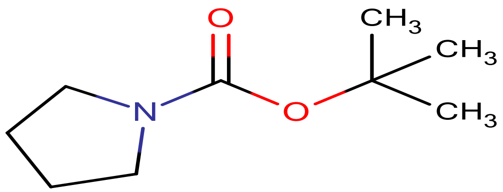	1-BOC-PYRROLIDINE	171.2
216	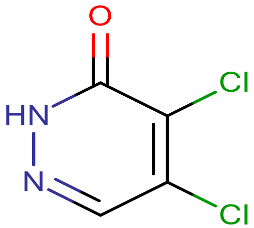	4,5-DICHLOROPYRIDAZIN-3[2H]-ONE	165.0
217	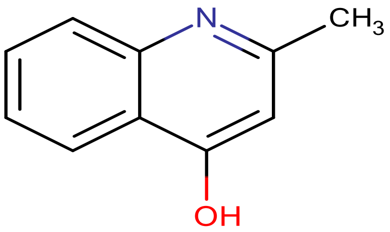	4-HYDROXY-2-METHYLQUINOLINE	159.2
218	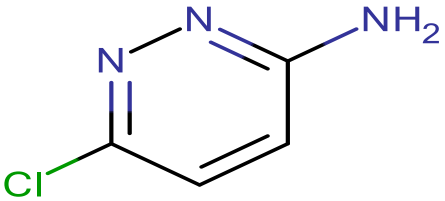	3-AMINO-6-CHLOROPYRIDAZINE	129.5

219	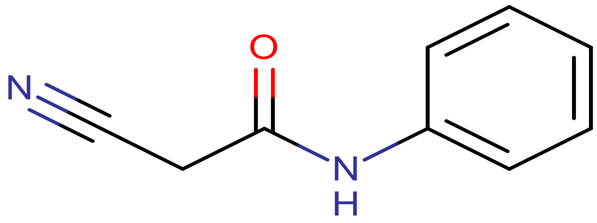	2-CYANOACETANILIDE	160.2
220	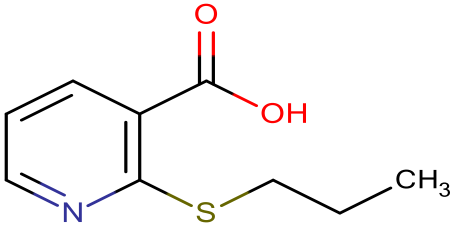	2-(N-PROPYLTHIO)NICOTINIC ACID	197.3
221	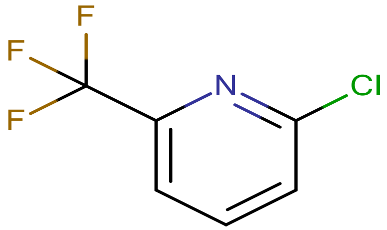	2-CHLORO-6-(TRIFLUOROMETHYL)PYRIDINE	181.5
222	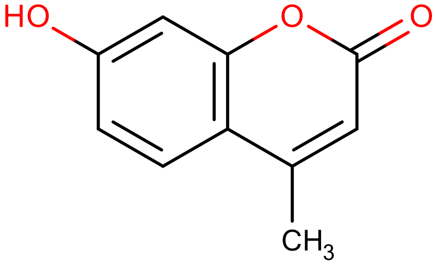	4-methylumbelliferone	176.2

223	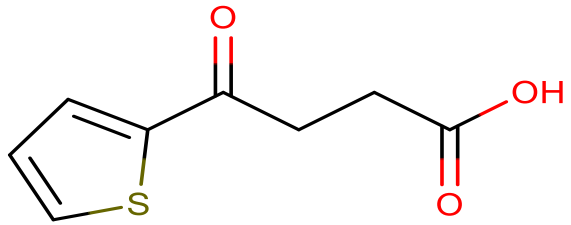	3-(2-THENOYL)-PROPIONIC ACID	184.2
224	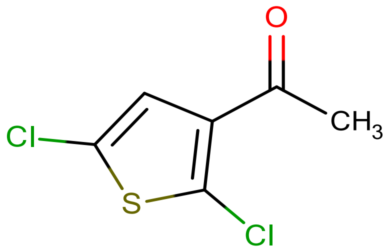	3-ACETYL-2,5-DICHLOROTHIOPHENE	195.1
225	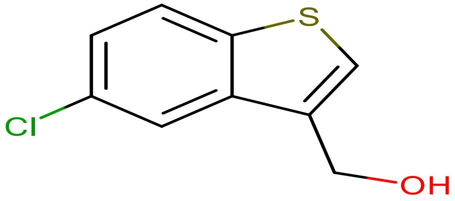	(5-CHLORO-1-BENZOTHIOPHEN-3-YL)METHANOL	198.7
226	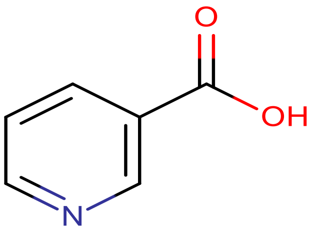	NICOTINIC ACID	123.1

227	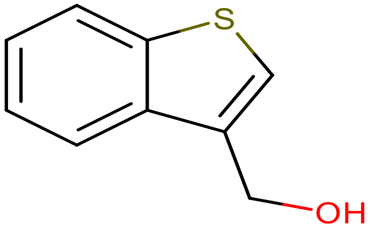	1-BENZOTHIOPHEN-3-YLMETHANOL	164.2
228	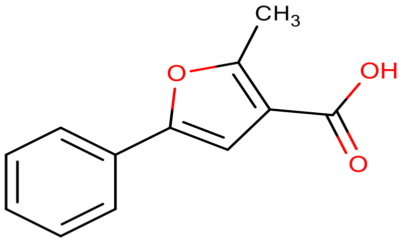	2-METHYL-5-PHENYLFURAN-3-CARBOXYLIC ACID	202.2
229	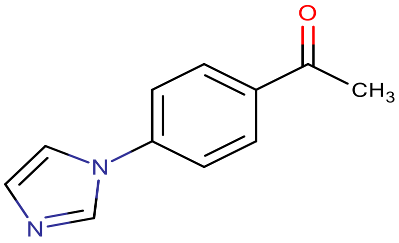	4'-(IMIDAZOL-1-YL)ACETOPHENONE	186.2
230	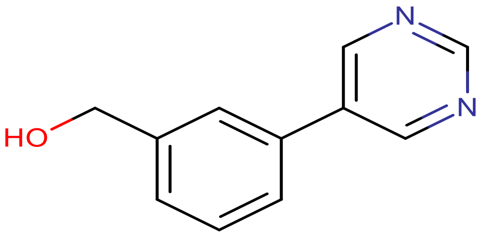	[3(5-Pyrimidinyl)phenyl]methanol	186.2

231	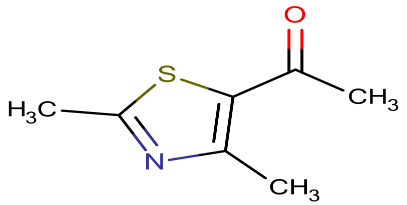	5-acetyl-2,4-dimethylthiazole	155.2
232	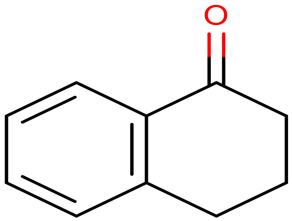	A-TETRALONE	146.2
233	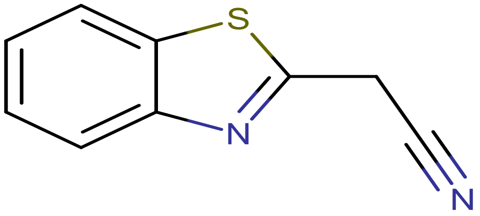	BENZOTHIAZOL-2-YLACETONITRILE	174.2
234	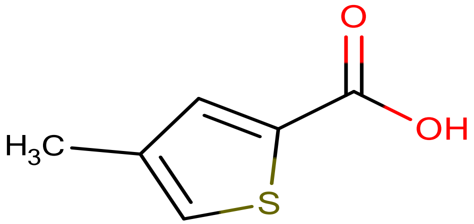	4-METHYLTHIOPHENE-2-CARBOXYLIC ACID, 97%	142.2

235	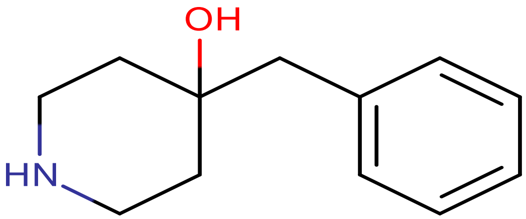	4-BENZYL-4-HYDROXYPIPERIDINE	191.3
236	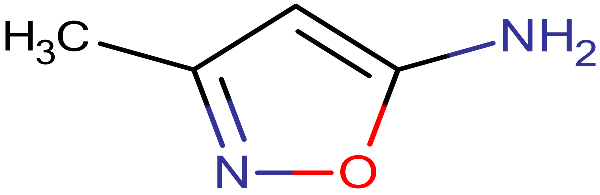	5-AMINO-3-METHYLISOXAZOLE	98.1
237	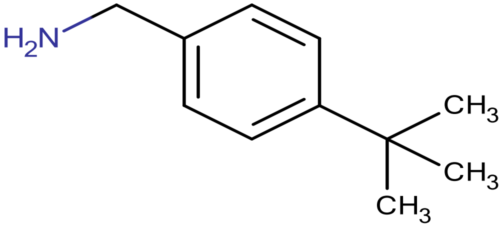	4-(T-BUTYL)-BENZYLAMINE	163.3
238	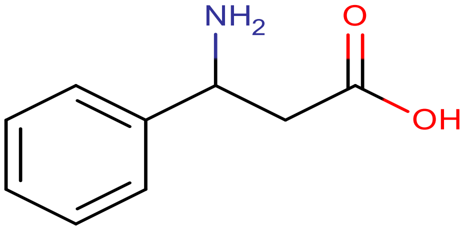	3-Amino-3-phenylpropanoic acid	165.2

239	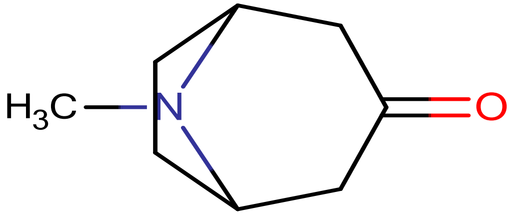	TROPINONE	139.2
240	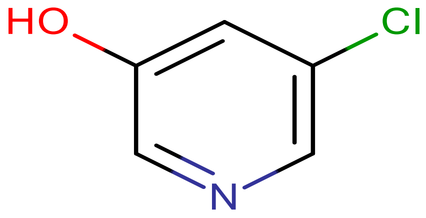	5-CHLORO-3-HYDROXYPYRIDINE, 99%	129.5
241	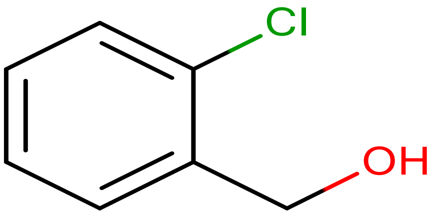	2-CHLOROBENZYL ALCOHOL	142.6
242	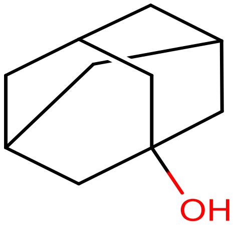	1-ADAMANTANOL	152.2

243	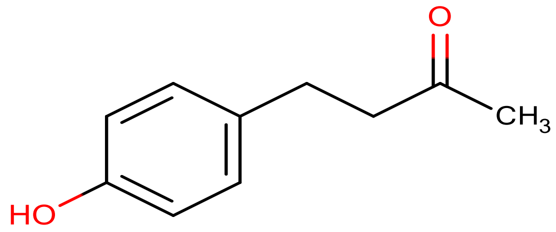	4-(4-HYDROXYPHENYL)-2-BUTANONE	164.2
244	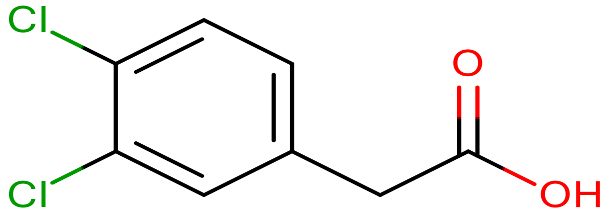	3,4-DICHLOROPHENYLACETIC ACID	205.0
245	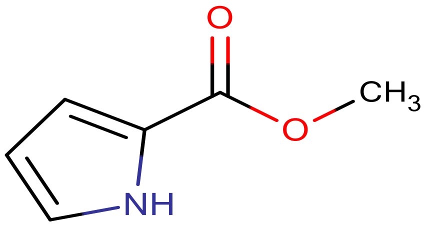	METHYL 2-PYRROLECARBOXYLATE	125.1

246	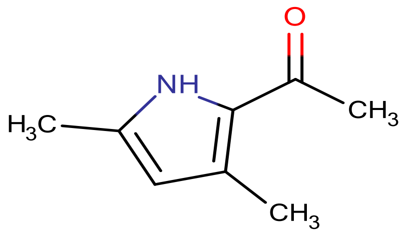	1-(3,5-dimethyl-1H-pyrrol-2-yl)ethanone	137.2
247	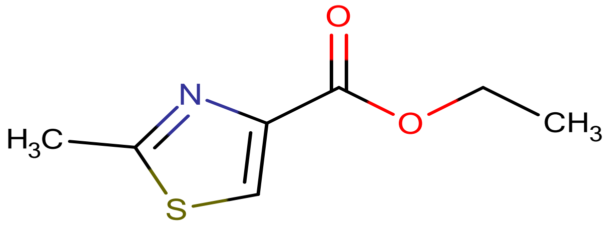	ETHYL 2-METHYLTHIAZOLE-4-CARBOXYLATE	171.2
248	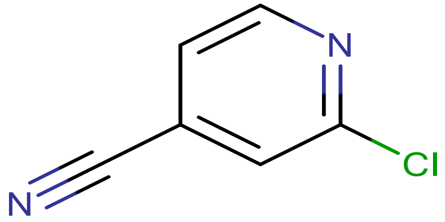	2-CHLORO-4-CYANOPYRIDINE	138.6
249	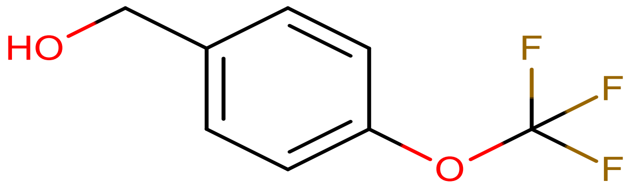	p-Trifluoromethoxy benzyl alcohol	192.1

250	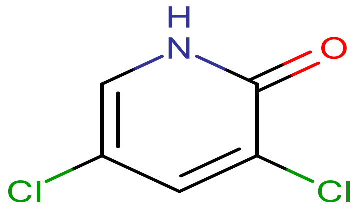	3,5-DICHLORO-2-PYRIDONE	164.0
251	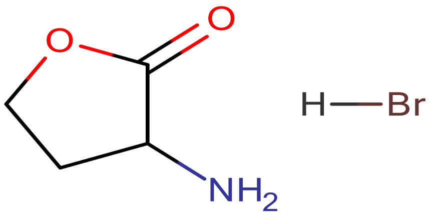	(+/-)-ALPHA-AMINO-GAMMA-BUTYROLACTONE HYDROBROMIDE	182.0
252	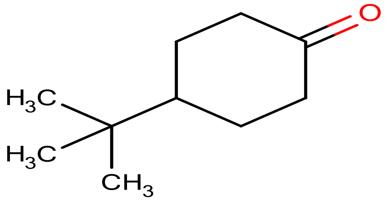	4-tert-BUTYLCYCLOHEXANONE	154.3
253	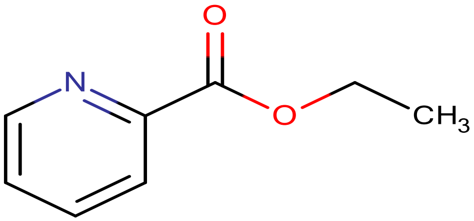	ETHYL PICOLINATE	151.2

254	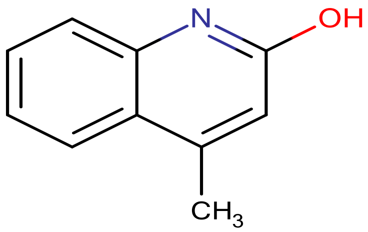	2-HYDROXY-4-METHYLQUINOLINE	159.2
255	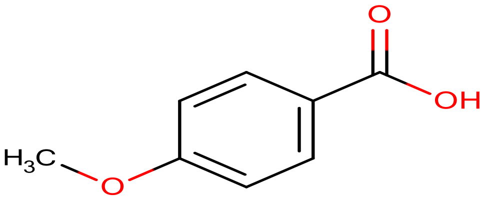	P-ANISIC ACID	152.1
256	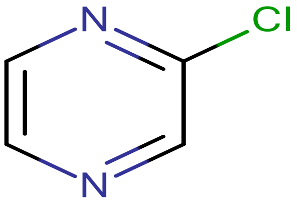	CHLOROPYRAZINE	114.5
257	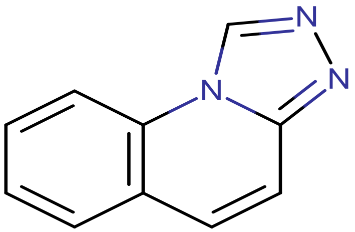	1,2,4-TRIAZOLO[4,3-A]QUINOLINE, 99%	169.2

258	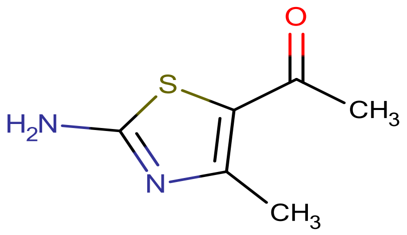	5-ACETYL-2-AMINO-4-METHYLTHIAZOLE	156.2
259	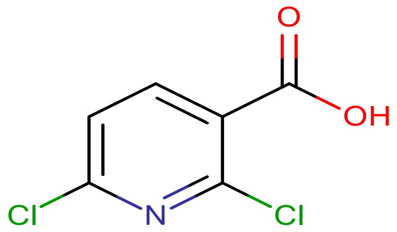	2,6-DICHLOROPYRIDINE-3-CARBOXYLIC ACID	192.0
260	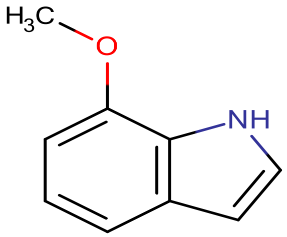	7-Methoxyindole	147.2
261	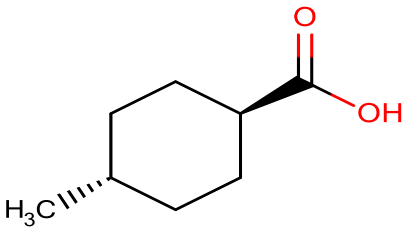	TRANS-4-METHYL-1-CYCLOHEXANECARBOXYLIC ACID	142.2

262	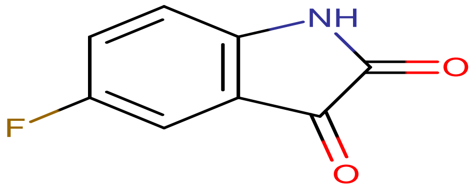	5-fluoroisatin	165.1
263	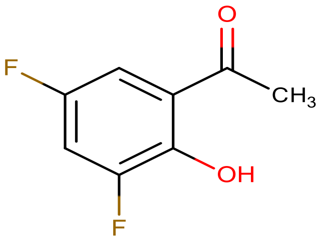	1-(3,5-DIFLUORO-2-HYDROXYPHENYL)ETHAN-1-ONE	172.1
264	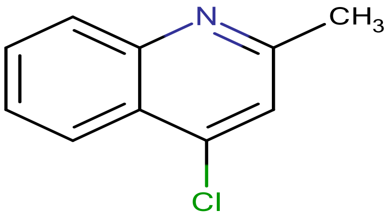	4-Chloro-2-methylquinoline	177.6
265	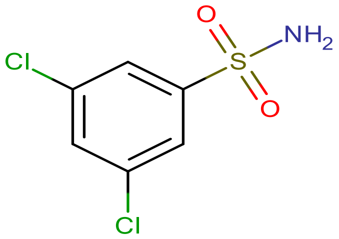	3,5-DICHLOROBENZENESULFONAMIDE	226.1

266	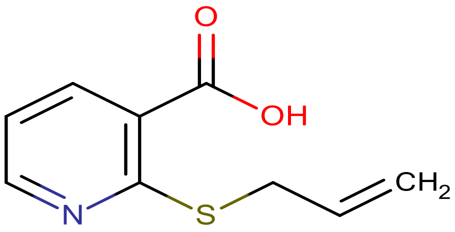	2-(ALLYLTHIO)NICOTINIC ACID	195.2
267	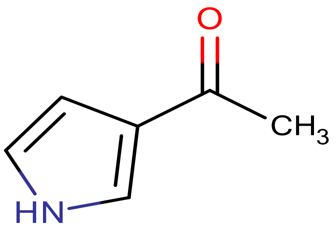	1-(1H-PYRROL-3-YL)ETHAN-1-ONE	109.1
268	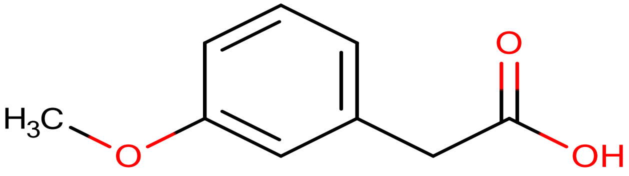	3-Methoxyphenyl acetic acid 99.5%	166.2
269	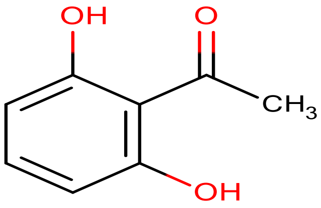	2',6'-Dihydroxyacetophenone	152.1

270	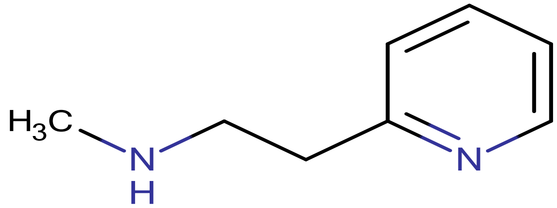	2-(B-METHYLAMINOETHYL)-PYRIDINE	136.2
271	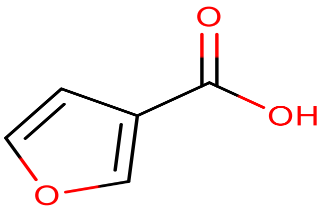	3-FUROIC ACID	112.1
272	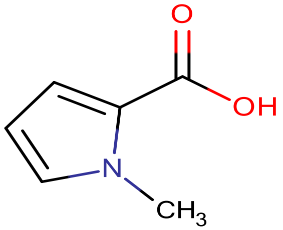	1-METHYL-2-PYRROLECARBOXYLIC ACID	125.1
273	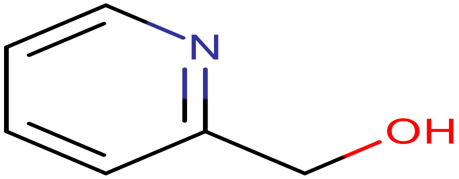	2-PYRIDYLCARBINOL	109.1

274	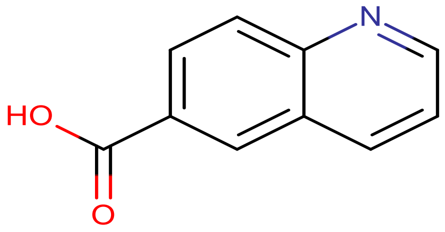	6-QUINOLINECARBOXYLIC ACID	173.2
275	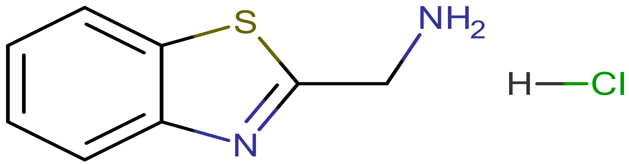	1,3-BENZOTHIAZOL-2-YLMETHYLAMINE HYDROCHLORIDE	200.7
276	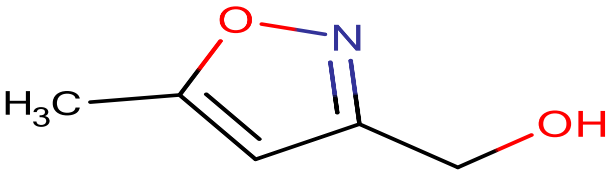	(5-METHYL-3-ISOXAZOLYL)METHANOL	113.1
277	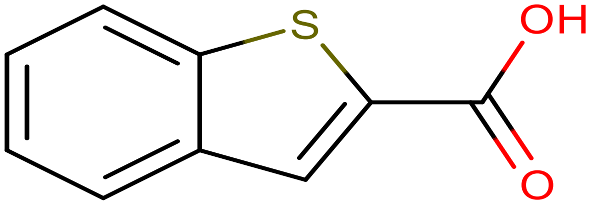	BENZO[B]THIOPHENE-2-CARBOXYLIC ACID	178.2

278	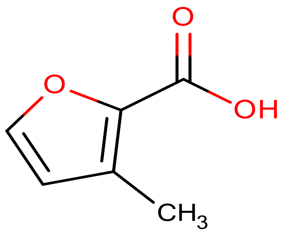	3-METHYL-2-FUROIC ACID	126.1
279	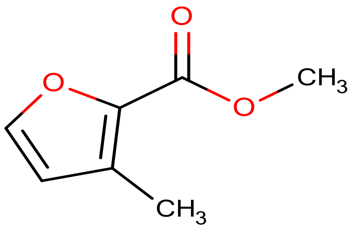	Methyl 3-methyl-2-furoate	140.1
280	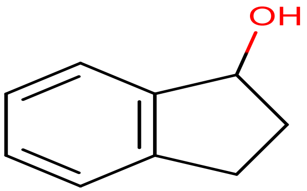	1-INDANOL	134.2
281	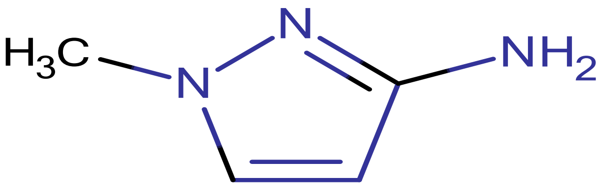	1-methyl-1h-pyrazol-3-amine	97.1

282	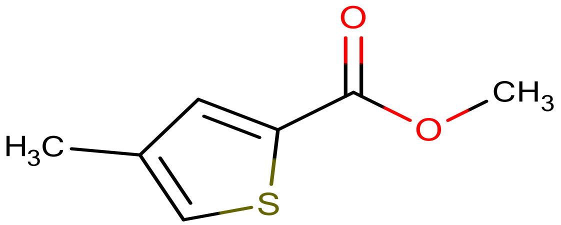	Methyl 4-methylthiophene-2-carboxylate	156.2
283	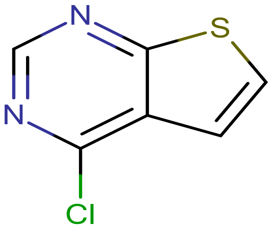	4-CHLOROTHIENO[2,3-D]PYRIMIDINE	170.6
284	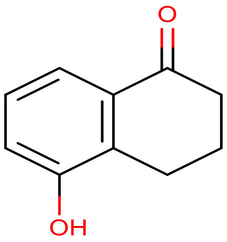	5-HYDROXY-1-TETRALONE	162.2

285	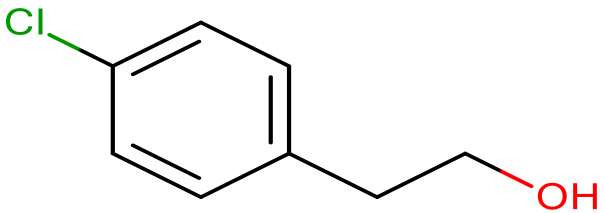	4-CHLOROPHENETHYL ALCOHOL	156.6
286	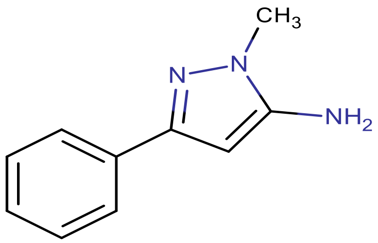	5-AMINO-1-METHYL-3-PHENYLPYRAZOLE	173.2
287	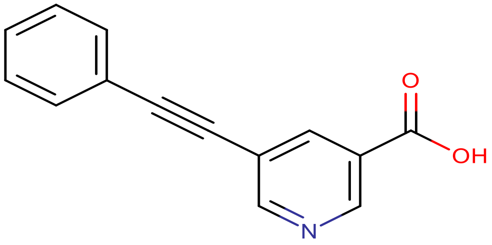	5-(2-PHENYLETH-1-YNYL)NICOTINIC ACID	223.2

288	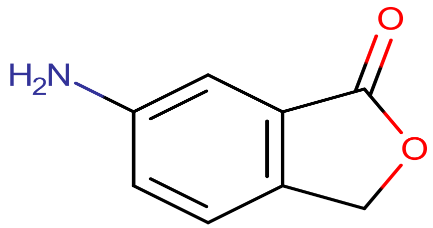	6-Aminophthalide 95%	149.1
289	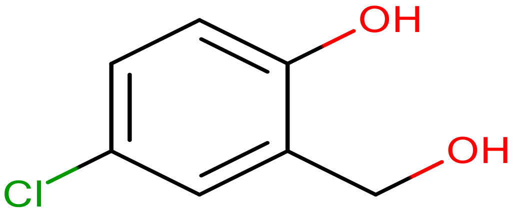	5-CHLOROSALICYL ALCOHOL	158.6
290	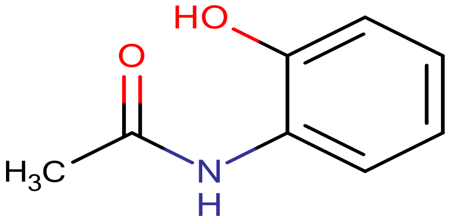	2-ACETAMIDOPHENOL	151.2

291	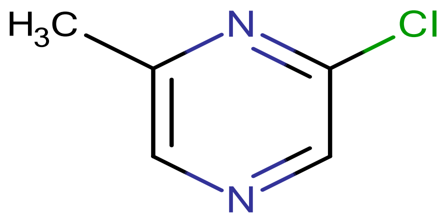	2-Chloro-6-methylpyrazine	128.6
292	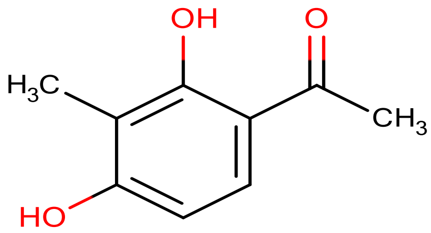	2',4'-DIHYDROXY-3'-METHYLACETOPHENONE	166.2
293	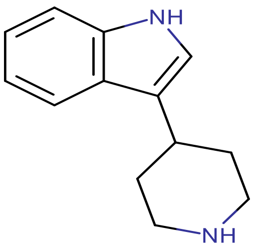	3-(PIPERIDIN-4-YL)-1H-INDOLE	200.3

294	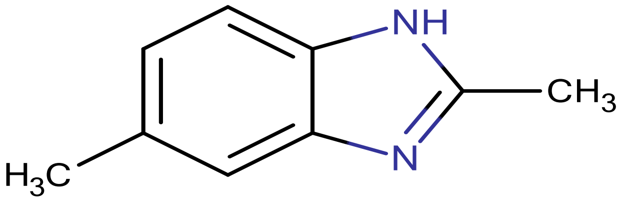	2,5-DIMETHYL-1H-BENZIMIDAZOLE	146.2
295	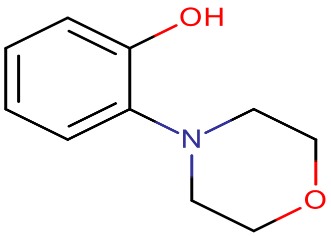	2-Morpholinophenol	179.2
296	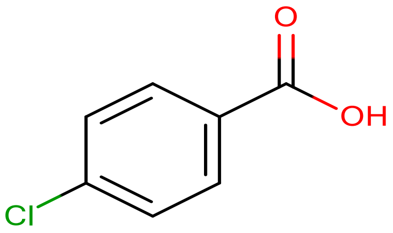	4-CHLOROBENZOIC ACID	156.6
297	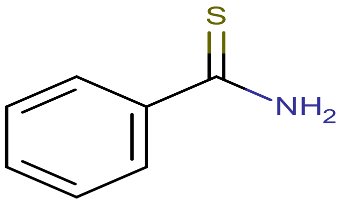	THIOBENZAMIDE	137.2

298	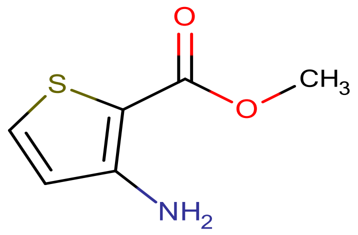	METHYL 3-AMINO-2-THIOPHENECARBOXYLATE	157.2
299	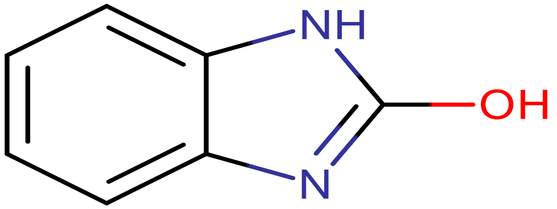	2-HYDROXYBENZIMIDAZOLE	134.1
300	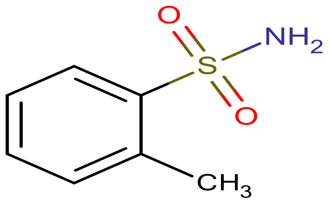	o-TOLUENESULFONAMIDE	171.2
301	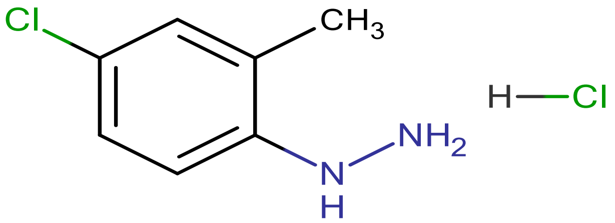	4-CHLORO-O-TOLYLHYDRAZINE HYDROCHLORIDE, 97+%	193.1

302	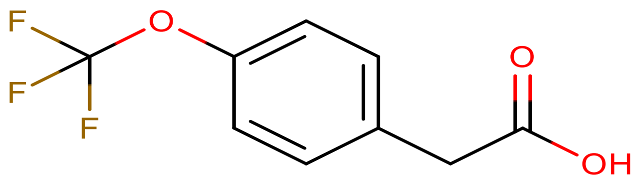	4-(TRIFLUOROMETHOXY)PHENYLACETIC ACID	220.1
303	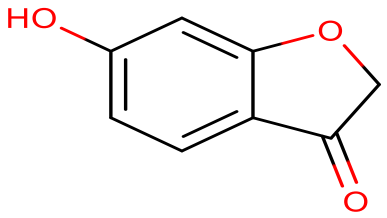	6-HYDROXY-2H-BENZOFURAN-3-ONE	150.1
304	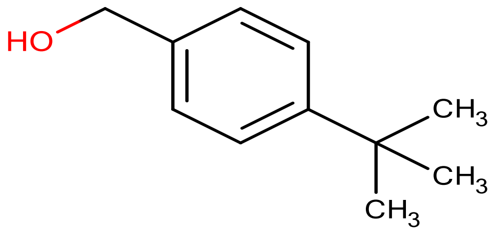	4-TERT-BUTYLBENZYL ALCOHOL	164.2
305	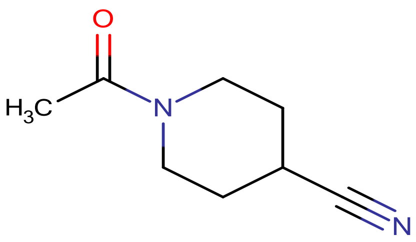	1-ACETYLPIPERIDINE-4-CARBONITRILE	152.2

306	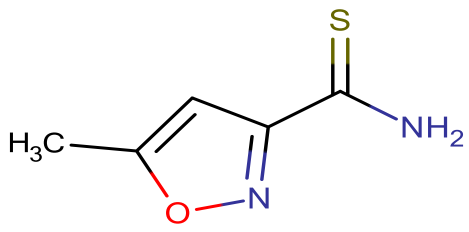	5-Methylisoxazole-3-carbothioamide	142.2
307	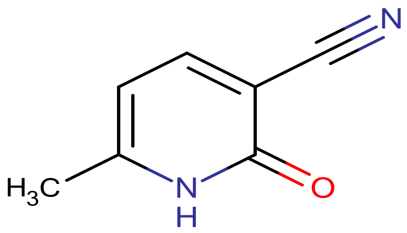	3-cyano-6-methyl-2(1H)-pyridinone 97%	134.1
308	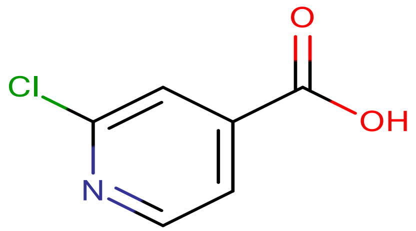	2-CHLOROISONICOTINIC ACID	157.6
309	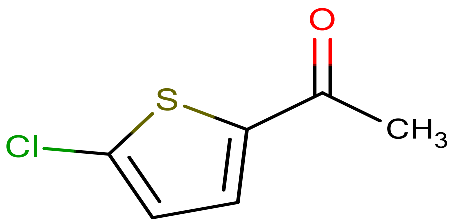	2-ACETYL-5-CHLOROTHIOPHENE	160.6

310	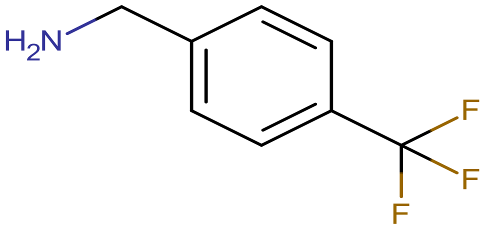	4-(TRIFLUOROMETHYL)BENZYLAMINE	175.2
311	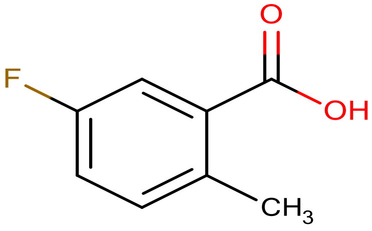	5-Fluoro-2-methyl benzoic acid	154.1
312	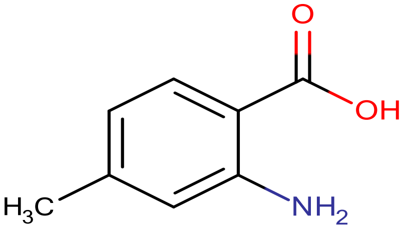	2-Amino-4-methylbenzoic acid	151.2
313	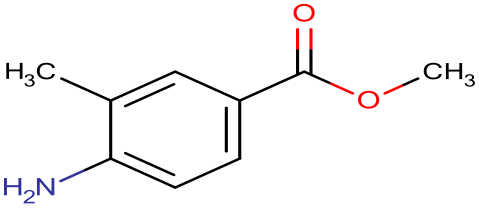	METHYL<br>4-AMINO-3-METHYLBENZOATE	165.2

314	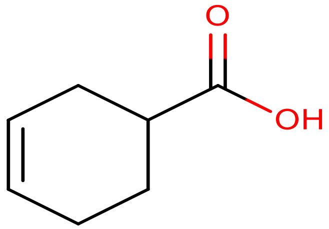	3-CYCLOHEXENE-1-CARBOXYLIC ACID	126.2
315	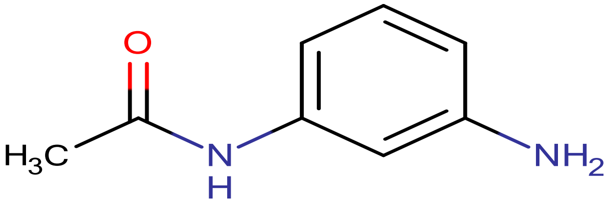	3'-AMINOACETANILIDE	150.2
316	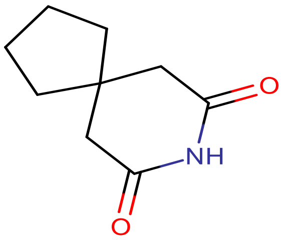	TETRAMETHYLENE GLUTARIMIDE	167.2

317	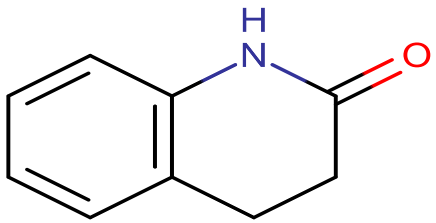	3,4-DIHYDRO-2(1H)-QUINOLINONE	147.2
318	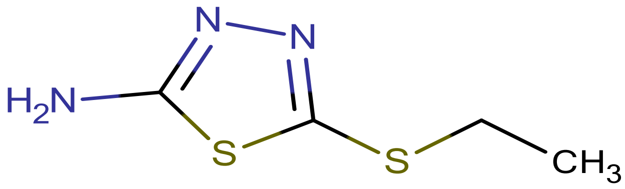	2-Amino-5-(ethylthio)-1,3,4-thiadiazole	161.3
319	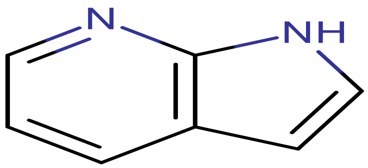	7-Azaindole	118.1

Supplementary Table S2

**Data Collection**	**TraE-1E6 complex**	**TraE-4H10 complex**	**TraE-105055 complex**	**TraE-239852 complex**
Wavelength (Å)	0.977	0.977	0.977	0.977
Resolution range (Å)	38.77 - 2.55	41.16 - 2.79	39.48 - 2.52	41.65 - 2.619
Space group	C 2 2 21	C 2 2 21	C 2 2 21	C 2 2 21
Unit cell parameters a (Å), b (Å), c (Å), α = β = γ = 90°	111.318, 123.766, 108.076	111.799, 124.116, 109.993	112.911, 125.535, 110.453	112.39, 124.089, 109.914
Total reflections	151 997	40 920	144 542	138 072
Unique reflections	38 437	15 838	26 767	23 012
Multiplicity	6.1	2.6	5.4	6.0
Completeness (%)	96.39	81.59	99.98	99.42
Mean I/sigma(I)	2.5	4.09	5.36	5.91
Wilson B-factor	41.88	67.50	57.14	44.50
R-work	0.2247	0.2980	0.2998	0.2167
R-free	0.2806	0.3569	0.3562	0.2740
Number of non-hydrogen atoms	4 688	4 660	4 621	4 654
Macromolecules	4 569	4 628	4 597	4 493
Ligands	16	10	14	14
Water	103	22	10	147
Protein residues	559	567	563	557
RMS bonds (Å)	0.008	0.006	0.004	0.006
RMS angles (°)	0.78	0.76	0.68	0.95
Ramachandran favored (%)	94.74	95.92	95.14	97.06
Ramachandran allowed (%)	5.08	3.72	4.68	2.75
Ramachandran outliers (%)	0.18	0.35	0.18	0.18
Clashscore	3.88	7.22	26.75	12.59
Average B-factor ($Å^2$)	61.90	65.80	75.00	54.90
**PDB number**	**5WIC**	**5WII**	**5WIO**	**5WIP**

Supplementary legends

Supplementary Figure 1. Fragment screening using DSF. *(A-F)* Effects of 505 fragments (5 mM final concentration) on TraE melting temperatures (T_m). Differences of T_m between fragments and a DMSO control are shown (ΔT_m). Positive shifts higher than 1°C (two times higher than the standard deviation of the DMSO control) were considered as indications for binding (black dashed line).

Supplementary Figure 2. Chemical formulas of binding fragments.

Supplementary Figure 3. Docking of small molecules derived from fragments 1E6 and 4H10. *(A)* Chemical formulas of small molecules derived from fragments 1E6 and 4H10. *(B-C)* Predicted binding of small molecules on TraE after docking with AutoDockVina software.

Supplementary Figure 4. Quantification of the formation of DSS-dependent cross-linking products of TraE. SDS-PAGE showing the cross-linking products of TraE (indicated by arrow) in the absence and in the presence (0 - 1.6 mM) of (A) 239852, (B) 105055, and (C) combination of 105055 and 239852 .

Supplementary Figure 5. *In vivo* characterization of fragments and small molecules effects on transfer of the unrelated plasmid RP4. Conjugation assays between plasmid RP4-carrying donor strain FM433 and plasmid-free recipient WL400 were conducted in the presence of 50 µM TraE-binding fragment and small molecules. The numbers of colony-forming units compared with a control experiment in the absence of the small molecules are shown; data represent averages and S.E. (error bars) of three biological replicate cultures.

Supplementary Table 1. Library of fragments

Supplementary Table 2. Data collection and refinement statistics for TraE in complex with fragments, 1E6 and 4H10, and small molecules, 105055 and 239852
